# Molecular innate immune programs of tumor-associated macrophages in immune checkpoint blockade resistance: a staged framework from suppressive circuitry to translational bottlenecks

**DOI:** 10.3389/fimmu.2026.1881958

**Published:** 2026-08-06

**Authors:** Yuzhe Huang, Junqi Zhang, Kaipeng Tu, Wenfeng Ye, Shaomin Zou, Zhe Tang, Cheng Zeng

**Affiliations:** 1 Department of Surgery, Center for Cancer Medicine, the Fourth Affiliated Hospital of School of Medicine, and International School of Medicine, International Institutes of Medicine, Zhejiang University, Yiwu, China; 2 Zhejiang Key Laboratory of Precision Diagnosis and Treatment for Lung Cancer, the Fourth Affiliated Hospital, Zhejiang University School of Medicine, Yiwu, China; 3 Department of Surgery, The Second Affiliated Hospital, Zhejiang University School of Medicine, Hangzhou, China

**Keywords:** immune checkpoint blockade, innate immunity, therapeutic resistance, tumor microenvironment, tumor-associated macrophages

## Abstract

Immune checkpoint blockade (ICB) has reshaped the treatment landscape for many malignancies, yet only a subset of patients achieve durable clinical benefit. Both primary and acquired resistance remain major barriers to long-term disease control. As central innate immune cells in the tumor microenvironment, tumor-associated macrophages (TAMs) have emerged as key contributors to this resistance. Beyond their well-recognized role in suppressing T-cell responses, TAMs influence stromal organization, metabolic stress, checkpoint ligand expression, and phagocytic activity within the TME. They also affect the intratumoral distribution and clearance of therapeutic antibodies. In this review, we discuss TAM-mediated resistance to ICB as a molecularly regulated innate immune process that is dynamic and adaptive. We consider how macrophages are recruited to tumors, how they are educated by local microenvironmental cues, and how innate immune signaling, metabolic regulation, and phagocytic checkpoints shape suppressive programs that limit the efficacy of ICB. This framework allows us to distinguish macrophage programs that recur across tumor types from those shaped by specific tissue contexts or tumor lineages, and to place diverse TAM subsets within a clinically relevant model of ICB resistance. We also review current TAM-directed therapeutic strategies, including CSF1/CSF1R blockade, inhibition of chemokine-dependent recruitment, metabolic and epigenetic reprogramming, targeting of phagocytosis checkpoints, and emerging delivery-based approaches. Although these strategies are supported by substantial mechanistic evidence, their clinical activity has so far been variable. This inconsistency likely reflects the marked heterogeneity and plasticity of TAMs, compensatory signaling pathways, spatial constraints within tumors, treatment-induced adaptation, toxicity, and the lack of robust biomarkers for patient selection. We propose that future TAM-directed therapies should move beyond broad macrophage depletion or nonspecific suppression. Instead, more precise approaches are needed—ones that are matched to dominant macrophage programs, informed by spatial and functional profiling, and guided by predictive biomarkers. Such strategies may help preserve beneficial innate immune macrophage functions while selectively disrupting the TAM states that sustain resistance to ICB.

## 1 Introduction

ICB has changed the treatment landscape for several cancers by interrupting inhibitory pathways such as PD-1/PD-L1 and CTLA-4, thereby restoring T-cell activity in a subset of patients (1, 2). Although some patients experience durable clinical responses, a substantial proportion exhibit primary or acquired resistance, highlighting the urgent need to understand the biological determinants of ICB efficacy (3, 4). The tumor microenvironment (TME) is central to this problem because malignant cells, stromal components, vasculature, and immune populations jointly determine whether antitumor immunity is sustained or suppressed (5–7). Thus, understanding ICB resistance requires moving from a T-cell-centered view toward a systems-level interpretation of the tumor microenvironment.

TAMs constitute a major myeloid component of the TME, but their biology cannot be reduced to a uniformly immunosuppressive identity (1, 8–14). In many tumor settings, macrophages are educated toward suppressive programs that blunt antitumor immunity and support ICB resistance. Yet this is not the only trajectory. Tissue-resident-like FOLR2+ macrophages, LYVE1+ perivascular macrophages, and interferon-primed TAMs with retained antigen-presenting capacity can preserve homeostatic, phagocytic, inflammatory, or immune-supportive functions, depending on tumor context, spatial localization, and local TME signals (13, 14). This functional diversity provides the rationale for TAM-directed strategies, including therapeutic depletion, recruitment blockade, and functional reprogramming in combination with ICB. Yet, despite strong mechanistic rationale and encouraging preclinical data, these approaches have produced heterogeneous and often modest clinical benefits. The more pressing question is therefore why clear immunosuppressive biology has not consistently translated into improved ICB responses in patients.

Across tumor types, TAM-mediated immunosuppression correlates with adverse clinical outcomes. In solid tumors such as breast, prostate, and colorectal cancer, enrichment of suppressive TAM programs is linked to therapy resistance, immune exclusion, and reduced survival (15–23). Spatial organization further dictates ICB efficacy: tumors with dense TAM clusters and sequestered T cells demonstrate poor ICB responsiveness compared to tumors with diffuse lymphocyte infiltration (16, 17). Moreover, TAM interactions with stromal elements, metabolic remodeling, and epigenetic regulation reinforce their suppressive phenotype, creating multiple barriers to effective immune activation (18–23). Therefore, a more useful framework should distinguish macrophage programs that are causal and therapeutically actionable from those that are merely correlative markers of an immunosuppressive tumor state.

**Figure 1:**
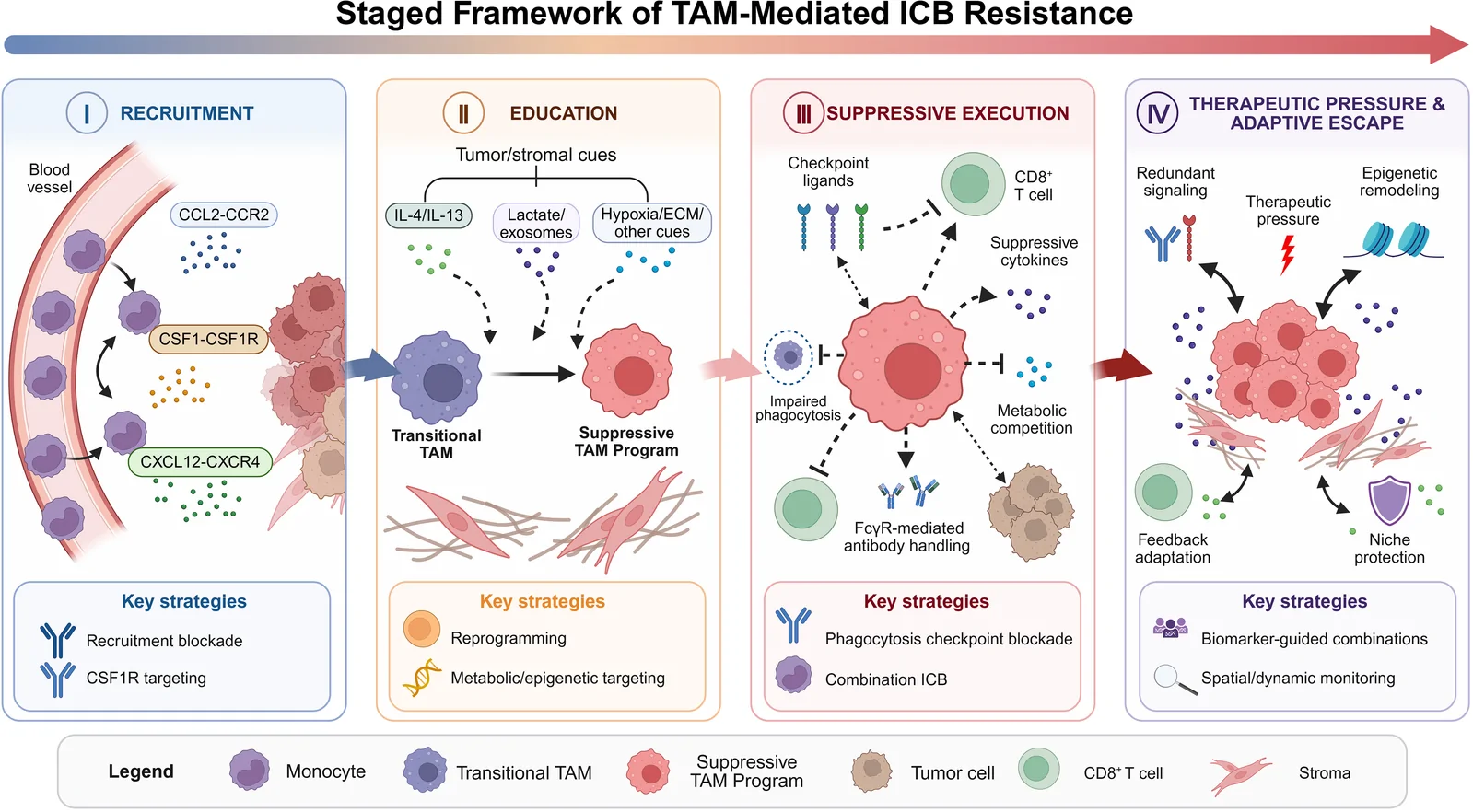
Staged framework of TAM-mediated ICB resistance and therapeutic entry points. TAM-mediated ICB resistance is summarized as a staged and adaptive process involving macrophage recruitment, intratumoral education, suppressive execution, and adaptive escape under therapeutic pressure. Representative TAM-directed entry points are mapped to each stage, highlighting how mechanism-matched strategies may interrupt macrophage-driven resistance.

In this review, we reinterpret TAM-mediated ICB resistance as a staged and adaptive process involving macrophage recruitment, intratumoral education, suppressive execution, and therapeutic escape after TAM-directed intervention. To retain biological continuity while avoiding a purely chronological description, the following sections organize these stages around four linked questions. First, how can TAM phenotypes, ontogenies, spatial niches, functional programs, and metabolic-epigenetic reprogramming pathways be integrated to define clinically actionable macrophage states? Second, how do educated TAMs execute immune suppression through cytokines, checkpoint ligands, metabolic remodeling, impaired phagocytosis, and antibody handling? Third, which therapeutic strategies can interrupt these programs without eliminating beneficial macrophage functions? Finally, why have many TAM-directed approaches shown inconsistent clinical benefit, and how can biomarker-guided, spatially informed trial designs improve translation? By structuring TAM biology around these linked stages, this review frames TAM-mediated ICB resistance as a molecular innate immune process involving macrophage recruitment, education, suppressive execution, and therapeutic adaptation. This staged framework and its corresponding TAM-directed therapeutic entry points are summarized in **Figure 1**.

## 2 Actionable heterogeneity and plasticity of tumor-associated macrophages

**Figure 2:**
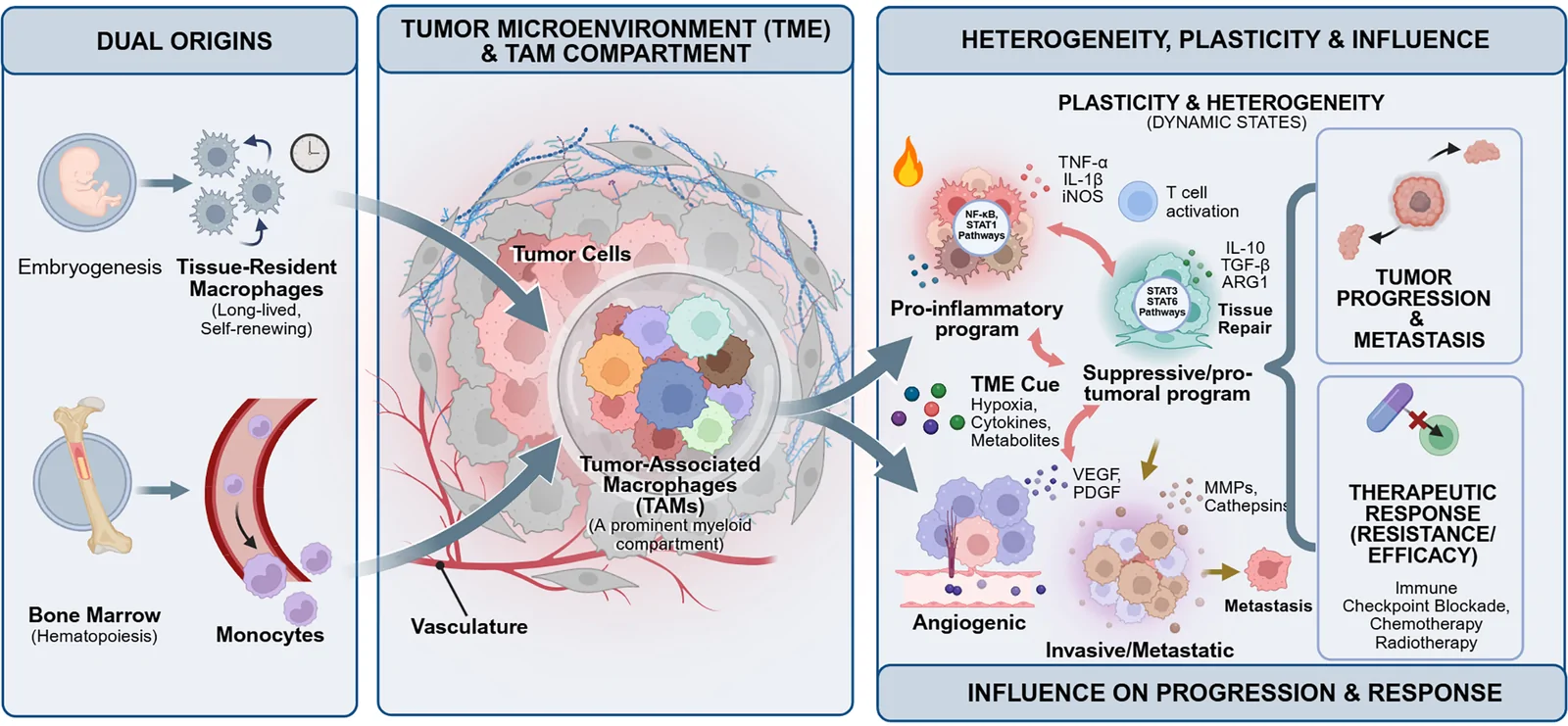
Heterogeneity and plasticity of tumor-associated macrophages (TAMs). TAMs arise from both tissue-resident macrophages and recruited monocytes. Within the TME, tumor-derived, stromal, metabolic, and inflammatory cues generate a continuum of macrophage states rather than fixed M1/M2 phenotypes.

The first two stages of TAM-mediated resistance to immune checkpoint blockade (ICB)—macrophage accumulation within tumors and their subsequent education by the tumor microenvironment—are closely intertwined. In practice, these processes are difficult to distinguish, because both recruited monocytes and tissue-resident macrophages rapidly adapt to suppressive tumor niches after encountering local tumor-, stromal-, metabolic-, and inflammation-derived signals. Although the M1/M2 framework remains useful as a conceptual shorthand, ICB-resistant tumors are better described by a continuum of hybrid TAM states that combine immunoregulatory, metabolic, inflammatory, and tissue-repair programs (14, 24). As illustrated in **Figure 2**, this heterogeneity arises from both resident and recruited macrophage pools and does not conform to fixed M1 or M2 identities. Instead, TAM phenotypes are shaped dynamically by cytokines, tumor-derived metabolites, hypoxia, extracellular matrix cues, and interactions with stromal and immune cells (24–27). Consistent with this view, single-cell and spatial transcriptomic analyses have shown that TAMs may co-express inflammatory mediators together with immunosuppressive molecules, enabling them to promote immune evasion while also supporting tissue remodeling, angiogenesis, and tumor progression (24, 25). Therefore, the key question is no longer whether TAMs can be assigned to classical M1 or M2 categories, but which hybrid macrophage programs are functionally required for ICB resistance and can be therapeutically targeted. Single-cell and multi-omic profiling has moved TAM biology beyond the binary M1/M2 model toward a program-based framework. Across tumors, macrophages can adopt recurrent interferon-primed, regulatory, inflammatory, lipid-associated, angiogenic, tissue-resident-like, and proliferating programs (12–14, 28, 29). These are best understood as functional states rather than fixed cell types. Interferon-primed TAMs may co-express chemokines, antigen-presentation machinery, and checkpoint ligands, giving them context-dependent roles in immune activation or exhaustion. Regulatory and lipid-associated programs can support complement-, lipid-, APOE-, TREM2-, and SPP1-associated suppressive niches, while inflammatory programs may amplify cytokine networks that sustain additional immunosuppressive cells. Angiogenic TAMs contribute to vascular remodeling, hypoxia, and immune exclusion, and proliferating TAMs may maintain local macrophage pools during tumor progression or after therapeutic pressure. Tissue-resident-like states, including LYVE1+ and FOLR2+ macrophages, may instead preserve homeostatic, phagocytic, or antigen-presenting functions in selected contexts. This heterogeneity makes broad “M2 macrophage” elimination an imprecise therapeutic concept. A more clinically useful approach is to define which TAM programs sustain ICB resistance in a given setting and which macrophage subsets should be spared or restored.

TAM heterogeneity also includes transcriptionally distinct and spatially restricted macrophage programs that are associated with clinical outcomes and treatment resistance, as summarized in **Table 1**. Additional representative TAM subsets and spatial patterns are listed in **Supplementary Table 1**. Importantly, however, the expanding catalogue of TAM populations should not be interpreted as a simple inventory of independent resistance mechanisms. For example, SPP1+ TAMs, C1q+ TAMs, TREM2+ macrophages, TREM-1+ macrophages, and USP14+ subsets have been linked, in different tumor types, to immune suppression, CD8+ T-cell dysfunction, hypoxic localization, recruitment of myeloid-derived suppressor cells, and resistance to ICB or targeted therapy (25, 30–37). Rather than representing entirely separate biological entities, these populations may reflect recurring macrophage programs that converge on several core functions, including stromal remodeling, immune silencing, metabolic adaptation, and therapy escape. This perspective moves the field beyond descriptive TAM taxonomy and toward a more functional framework, in which macrophage states are prioritized according to their clinical association, mechanistic contribution, and therapeutic tractability. TAMs are not terminally fixed; their plasticity allows tumors to reinforce immune escape through locally imposed survival, metabolic, and stromal cues. Malignant and stromal cells can provide instructive cues such as IL-4, HSP90α-loaded extracellular vesicles, VEGF-dependent signals, and cIAP1/2-regulated survival pathways, thereby sustaining macrophage-supported resistance niches (24, 41–43). Importantly, the same environmental dependence creates a therapeutic opening: disrupting the cues that maintain suppressive macrophage states can redirect TAMs toward immunostimulatory or phagocytic functions, as illustrated by VEGF inhibition combined with imipramine or cIAP1/2 antagonism (42, 43). Therefore, TAM plasticity should be viewed simultaneously as a mechanism of adaptive resistance and as a vulnerability that can be therapeutically exploited when the dominant educating signal is identified.

**Table 1:** Actionable TAM states and spatial programs in ICB resistance.

TAM program	Representative examples	Main implication	Ref.
Suppressive macrophage modules	SPP1+ TAMs, C1q+ TAMs, TREM2+ TAMs	Recurrent TAM states linked to immune suppression and ICB resistance	(25, 30–34)
Hypoxia-associated TAM programs	TREM-1+ TAMs	Support Treg recruitment and resistance in hypoxic niches	(35)
Therapy-resistant TAM subsets	Rps19/C5aR1-associated TAMs	May persist after treatment and drive adaptive resistance	(36)
Myeloid-recruiting TAM states	USP14+ TAMs	Promote MDSC recruitment and suppressive myeloid remodeling	(37)
Stromal–myeloid neighborhoods	SPP1+ TAM–CAF structures, IL4I1+ TAM–TDO2+ fibroblast niches	Spatial organization determines immune exclusion	(25, 38)
Physical immune barriers	Perivascular TAMs, macrophage-coated tumor clusters	Block T-cell infiltration despite immune activation	(39, 40)

Ultimately, TAM heterogeneity is architectural as well as transcriptional. Spatial transcriptomic analyses show that TAMs do not scatter randomly but form organized “neighborhoods” with cancer-associated fibroblasts, endothelial cells, tumor cells, and exhausted lymphocytes, thereby shaping local immune accessibility (25, 38–40). IL4I1+ TAMs interacting with TDO2+ myofibroblasts, SPP1+ TAMs embedded within stromal–myeloid structures, perivascular macrophages that support angiogenesis and relapse, and macrophage-coated tumor clusters that block immune infiltration all illustrate how macrophage location modifies macrophage function (25, 38–40). Tumor-intrinsic programs such as WNT11, Tyro3, and CDK9 may further reinforce macrophage-rich immune-exclusion barriers, suggesting that oncogenic signaling and TAM-mediated suppression function as mutually reinforcing layers of immune escape rather than separable processes (44, 45). Consequently, the therapeutic relevance of a TAM subset cannot be inferred from marker expression alone; it must be interpreted together with spatial position, neighboring cell types, and contribution to local immune exclusion.

TAM heterogeneity is therefore most useful when it is linked to function, therapeutic vulnerability, and clinical outcome. A TAM state becomes clinically meaningful only when three conditions are met: it is enriched in nonresponders, its perturbation restores antitumor immunity in relevant models, and it can be targeted without eliminating beneficial macrophage functions such as antigen presentation, phagocytosis, or antibody-dependent tumor clearance. This distinction separates macrophage signatures associated with an immunosuppressive TME from programs that actively sustain ICB resistance (46). These criteria link macrophage recruitment and education to the downstream suppressive programs through which TAMs sustain ICB resistance.

Current evidence supports a graded, rather than uniform, interpretation of TAM subsets in ICB resistance. SPP1+ TAMs are among the best-supported populations functionally, with evidence extending beyond their recurrent enrichment in immune-excluded and ICB-resistant tumors. Across several settings, SPP1 perturbation has been associated with reduced suppressive macrophage activity and partial restoration of CD8+ T-cell function. Recent mechanistic data further showed that intracellular SPP1 interacts with TRIM21 to restrain SOCS1 ubiquitination, thereby limiting IFN-γ–STAT1–ISG antitumor signaling in TAMs (47). TREM2+ and TREM-1+ TAMs also have perturbation-based support, as blockade of these pathways improved antitumor immune activity or reversed checkpoint blockade resistance in experimental models (34, 35). By comparison, C1q+ TAMs, USP14+ TAMs, and several spatially resolved TAM neighborhoods remain supported primarily by transcriptomic, spatial, and clinical association studies, despite plausible mechanistic signals in selected reports. These populations should therefore be viewed as occupying different positions on an evidence continuum, rather than as equally validated causal drivers of ICB resistance.

## 3 Core mechanisms of TAM-mediated ICB resistance

**Figure 3:**
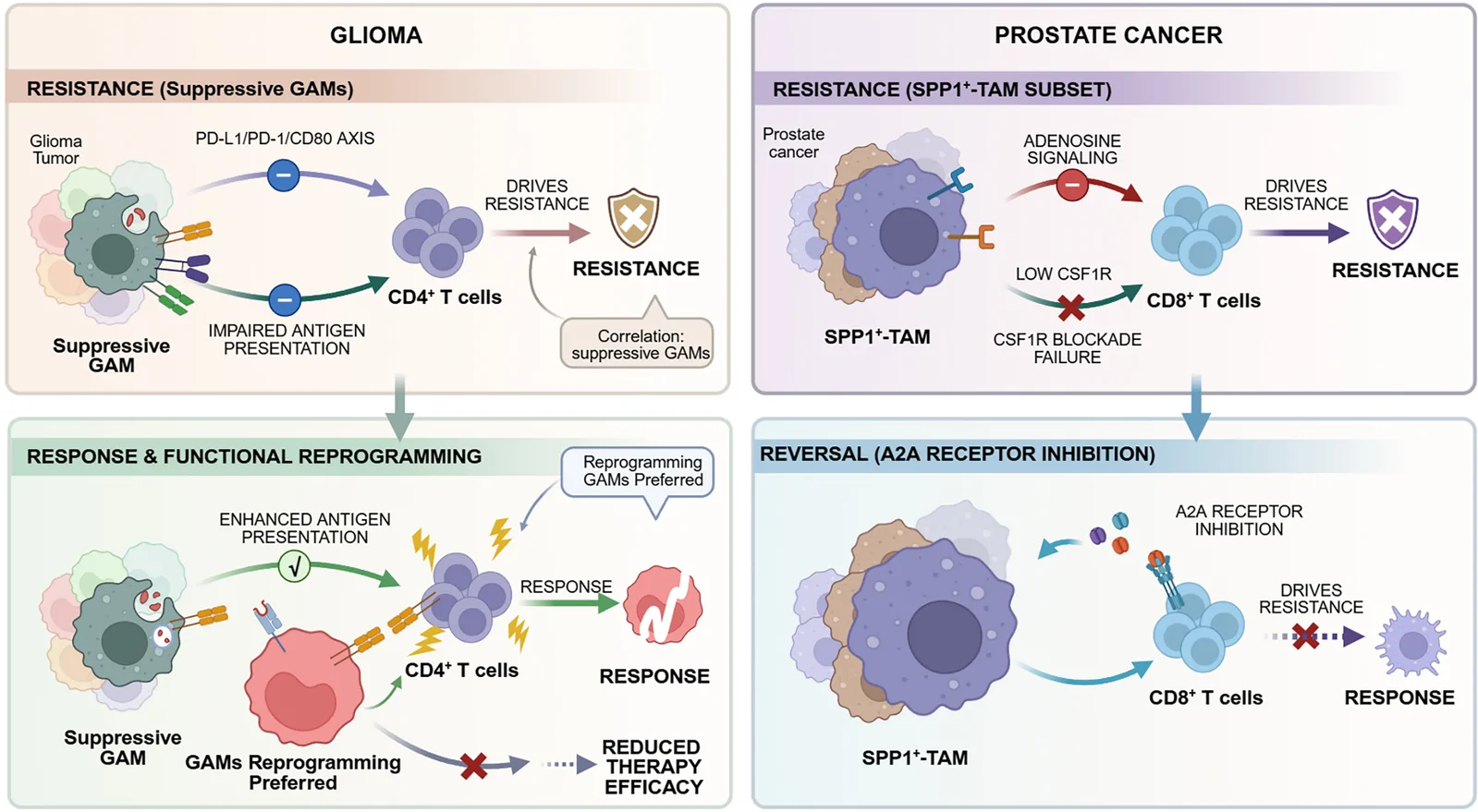
Representative TAM-mediated ICB resistance and reversal mechanisms in glioma and prostate cancer. In glioma, suppressive glioma-associated microglia/macrophages impair CD4+ T-cell responses through the PD-L1/PD-1/CD80 axis and defective antigen presentation, whereas macrophage reprogramming restores antigen presentation and response. In prostate cancer, SPP1+ TAMs drive resistance through adenosine signaling and low CSF1R-associated failure of CSF1R blockade, while A2A receptor inhibition restores CD8+ T-cell activity and reverses resistance.

After recruitment and local education, TAMs contribute to ICB resistance through several recurring mechanisms, including suppression of T-cell priming and activation, expression of alternative checkpoint ligands, metabolic competition with effector lymphocytes, impaired phagocytosis and antigen presentation, and Fc receptor–dependent handling of therapeutic antibodies. However, the upstream signals that dominate these programs vary substantially across tumor types and tissue environments. The major TAM-driven resistance mechanisms are summarized in **Table 2** and **Figure 3**. Importantly, TAM-mediated resistance is not restricted to effects on T cells. In glioma, for example, poor responsiveness to checkpoint blockade is closely associated with the polarization state of glioma-associated microglia and macrophages, where M2-like accumulation appears to represent an active immunosuppressive program rather than simply a lack of antitumor immunity (97). Outside the central nervous system, M2-polarized macrophages have been shown to weaken NK-cell cytotoxicity in prostate cancer (98), and macrophage–melanoma cell fusion has been proposed as a physical mechanism by which melanoma cells evade cytotoxic T-cell attack (99). The following subsections therefore discuss the major recurrent TAM-mediated resistance programs, with particular attention to how their contribution is shaped by tissue context, tumor genotype, spatial organization, and prior therapy.

**Table 2:** Core TAM-mediated resistance programs to ICB.

Resistance program	Representative mechanisms	Immune consequence	Ref.
TAM education and suppressive programming	IL-4, CAF-derived signals, hypoxia, acidosis, lactate	Drives suppressive and tissue-repair macrophage states	(24, 48–61)
Soluble suppressive circuits	Granulin, SPP1, CCL7, IL-34, IL-33	Excludes or exhausts CD8+ T cells and expands suppressive cells	(32, 62–70)
Checkpoint ligand expression	PD-L1, VISTA, Siglec-15, TIM-3, IDO-1	Directly restrains T-cell activation	(71–84)
Metabolic suppression	Lactate, kynurenine–AHR, adenosine–A2A, arginine depletion	Impairs T-cell fitness and reinforces suppressive TAM programs	(18, 85–89)
Stromal–myeloid coupling	CAF-derived C3, SAA, TSG-6, VEGFA/NRP2, M2BP	Creates immune-excluding niches	(5, 25, 30, 40, 90)
Impaired phagocytosis	CD47–SIRPα, CD24–Siglec-10/G, STC1, BCL9–BMP4	Blocks macrophage-mediated tumor clearance and antigen presentation	(91–95)
Antibody handling	FcγR-mediated antibody stripping or sequestration	Reduces effective ICB engagement	(96)

### 3.1 Macrophage education and suppressive TAM programs beyond M1/M2 polarization

**Figure 4:**
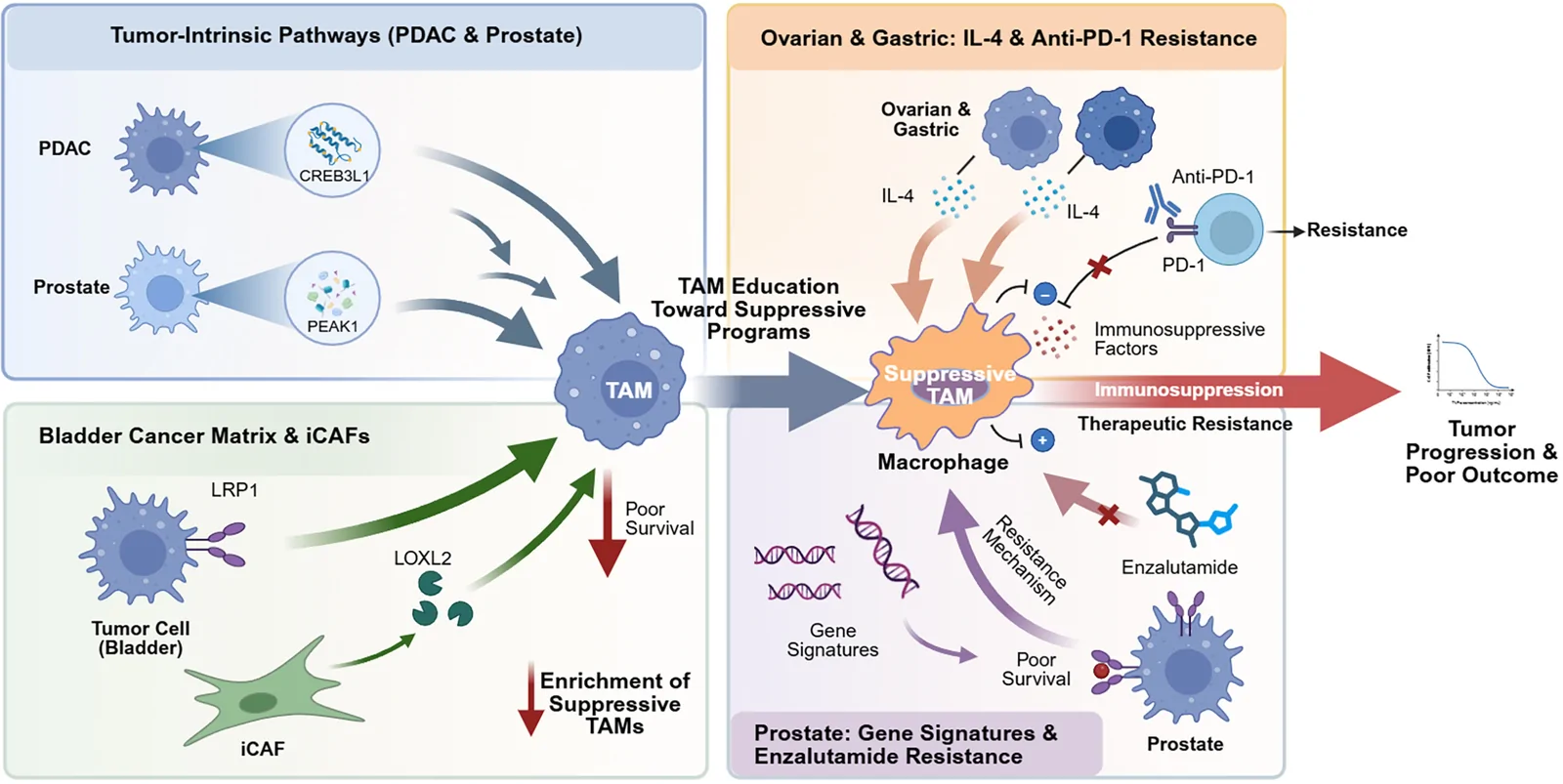
Tumor- and stroma-driven TAM education across cancer types. CREB3L1 and PEAK1 promote suppressive TAM education in pancreatic ductal adenocarcinoma and prostate cancer, respectively. Tumor-cell LRP1 and iCAF-derived LOXL2 enrich suppressive TAMs in bladder cancer. IL-4-dependent macrophage education contributes to anti-PD-1 resistance in ovarian and gastric cancers, while TAM-associated gene programs are linked to enzalutamide resistance and poor survival in prostate cancer.

Macrophage polarization is continuously shaped by tumor-intrinsic signals, stromal interactions, macrophage-intrinsic pathways, and metabolic pressures within the TME, as illustrated in **Figure 4**. Throughout this review, M1-like and M2-like terminology is used as operational shorthand for immunostimulatory- or suppressive-enriched macrophage programs, rather than as discrete, terminal, or mutually exclusive TAM categories. TAM states are stabilized by more than marker expression. Metabolic wiring and epigenetic regulation also help maintain macrophage function within the TME. Recent work has linked distinct macrophage activities to glycolytic remodeling, oxidative phosphorylation, fatty acid oxidation, lipid handling, mitochondrial remodeling, and nutrient-sensing pathways (100). Lactate can further imprint macrophage function through histone lactylation. In glioblastoma, glucose-driven histone lactylation enhances the immunosuppressive activity of monocyte-derived macrophages and has been linked to ICB response (101, 102). These findings support a continuum model of tumor macrophage polarization, in which functional states are metabolically and epigenetically reinforced rather than arranged along a binary M1/M2 axis. Rather than viewing M2-like polarization as a fixed endpoint, it is more useful to interpret it as an ongoing education process by which tumors impose suppressive, tissue-repair, and immune-excluding functions on macrophages. Context-specific routes into this state include CREB3L1, PEAK1, the LRP1–DLL4–Notch2–CCL2 axis, LOXL2-high inflammatory CAFs, tumor-derived IL-4, disrupted Cx43–cGAMP–STING signaling, MALT1, FKBP51s, GPSM1, PERK+ macrophage programs, GPR65-mediated acidosis sensing, lactate-driven histone lactylation, and methionine availability (24, 48–60). Across these diverse inputs, shifts toward suppressive, tissue-repair, lipid-associated, angiogenic, checkpoint ligand–expressing, or metabolically reinforced macrophage programs remain associated with ICB resistance, but these programs should be interpreted through their dominant ontogenic, metabolic, epigenetic, and spatial drivers rather than through the M1/M2 ratio alone (61, 100–102). Accordingly, the key question is whether a given polarization cue is a dominant and reversible driver of resistance in a specific tumor, or merely a downstream marker of an already suppressive microenvironment.

Together, these observations caution against treating macrophage-associated signatures as direct evidence of causality. Transcriptomic macrophage enrichment and functional TAM programs have been associated with anti-androgen resistance, enzalutamide-induced immunosuppression, poor-survival bladder cancer subtypes, basophil-associated macrophage enrichment, communication-hub TAMs in NSCLC, and hyperprogressive disease after ICB (103–108). Recent single-cell and pan-cancer analyses further support a program-based view of TAM heterogeneity that cannot be reduced to mutually exclusive M1 or M2 states (13, 28, 29). Although these signatures may be useful biomarkers, they do not automatically justify broad depletion of suppressive macrophages or nonspecific macrophage elimination. Their therapeutic value depends on whether the associated program is functionally validated and causally linked to resistance in a given tumor context, rather than merely reflecting a uniform M2-polarized phenotype.

### 3.2 Secretion of immunosuppressive cytokines and soluble factors

**Figure 5:**
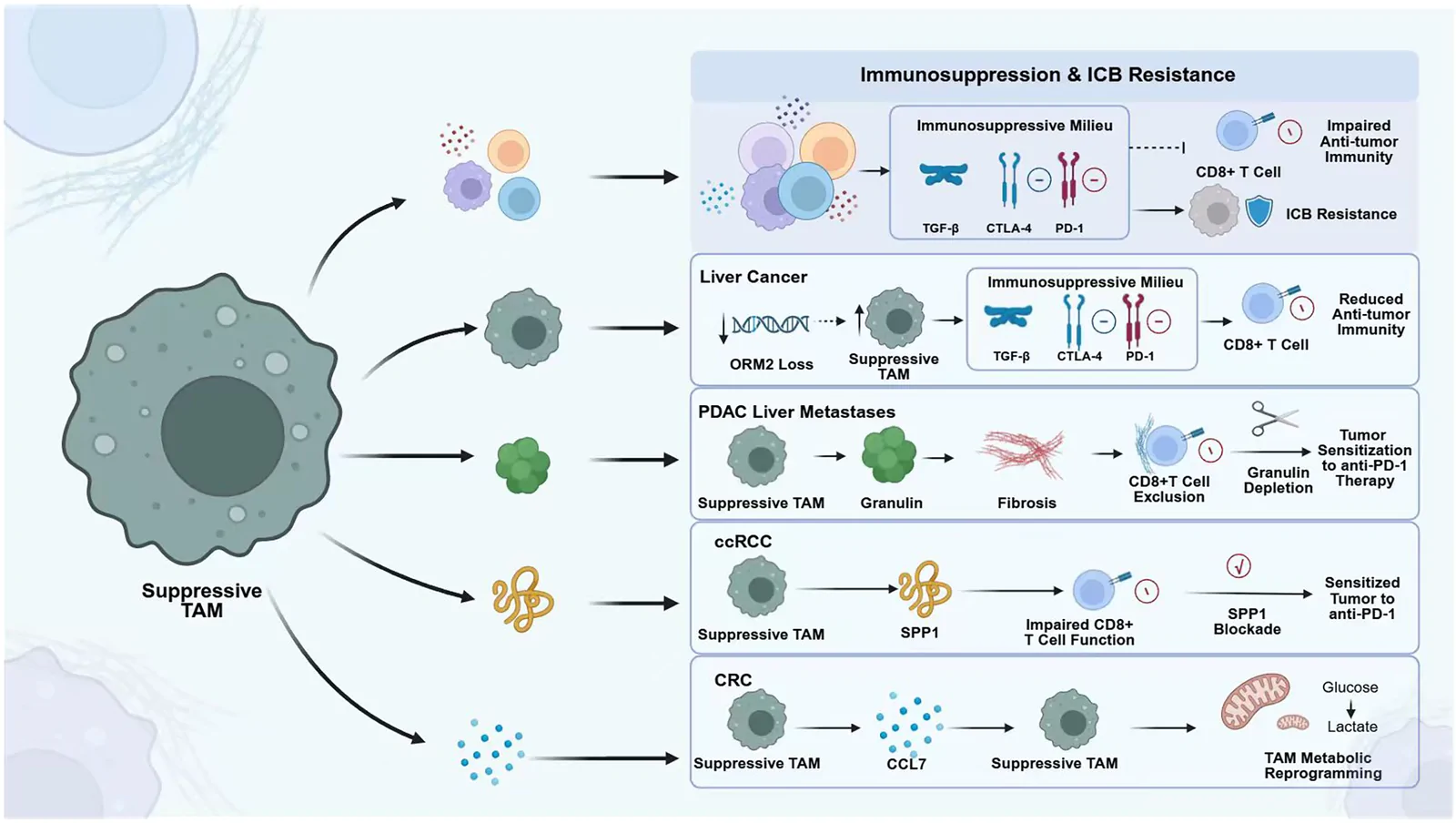
Soluble suppressive circuits driven by TAMs. Suppressive TAMs remodel the TME through cytokines, chemokines, extracellular vesicles, and other soluble mediators. These circuits impair CD8+ T-cell infiltration and effector function, thereby contributing to primary or acquired ICB resistance.

Polarized TAMs actively reshape the tumor microenvironment through a broad set of secreted mediators that weaken antitumor immunity (**Figure 5**). In liver cancer, reduced ORM2 expression is associated with increased TAM infiltration and an immunosuppressive milieu characterized by higher levels of TGF-β, CTLA-4, and PD-1 (109). The functional importance of these soluble factors has been supported by perturbation studies in several tumor settings. In PDAC liver metastases, for instance, macrophage-derived granulin drives fibrotic remodeling and limits CD8+ T-cell access to tumor lesions; depletion of granulin restores sensitivity to anti-PD-1 therapy (62). In ccRCC, SPP1+ TAMs impair CD8+ T-cell function through secretion of SPP1, and blockade of this pathway enhances the efficacy of anti-PD-1 treatment (32). In colorectal cancer, TAM-derived CCL7 acts in a dual manner: it supports macrophage metabolic fitness while also restricting CD8+ T-cell infiltration, and its inhibition shows synergy with anti-PD-L1 therapy (63). IL-34, produced by either tumor cells or macrophages, promotes the expansion of immunosuppressive CD163+ TAMs and has been linked to resistance to ICB, PARP inhibition, and radiotherapy (64–66). Similarly, the IL-4/IL-4R axis contributes to CD8+ T-cell dysfunction indirectly by promoting M2-like macrophage polarization (52). In C1q+ TAMs, FABP5–PPAR-γ-dependent fatty acid metabolism increases PD-L1 expression, and FABP5 inhibition enhances the response to anti-PD-1 therapy (33). Together, these findings suggest that soluble TAM-derived signals do not act as isolated suppressive molecules, but instead cooperate to reinforce immunosuppressive niches within tumors.

Crosstalk circuits further propagate suppression. In HCC, an IFN-γ–IL-1β–PIM2 axis between T cells, TAMs, and tumor cells drives resistance and is reversible by IL-1β or PIM2 blockade (67). In thyroid cancer, M2-derived extracellular vesicles deliver miR-21-5p to tumor cells, suppressing METTL3 and stabilizing CD70 to promote T-cell exhaustion (68). Tumor-derived exosomal PD-L1 inhibits T cells directly or induces PD-L1 on myeloid cells, causing systemic resistance (69). In liver metastases, tumor-derived IL17D polarizes macrophages to a CD206+ suppressive phenotype (45), while lymph node macrophage-derived IL-33 activates tumor-infiltrating Tregs (70). Thus, therapeutic strategies targeting soluble factors should be designed around the dominant suppressive circuit in each tumor rather than assuming that blockade of a single mediator will be broadly sufficient.

### 3.3 Expression of immune checkpoint ligands and myeloid checkpoint networks

**Figure 6:**
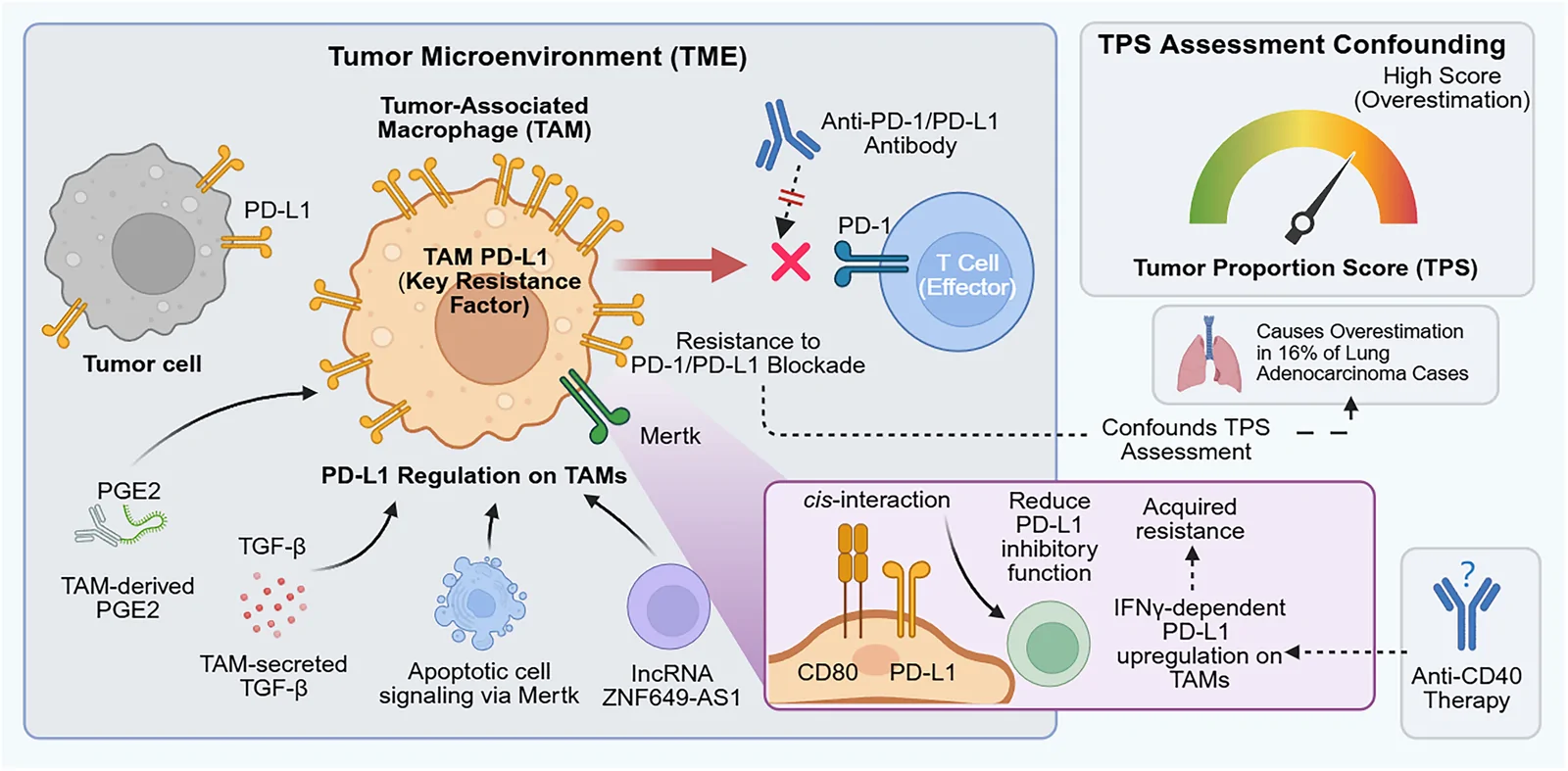
TAM PD-L1 regulation, ICB resistance, and tumor proportion score confounding. TAM PD-L1 suppresses PD-1+ effector T cells and can confound tumor proportion score assessment. PGE2, TGF-β, Mertk signaling, and lncRNA ZNF649-AS1 regulate TAM PD-L1. PD-L1–CD80 cis interaction reduces PD-L1 inhibitory function, whereas anti-CD40 therapy can induce IFN-γ-dependent PD-L1 upregulation and acquired resistance.

TAMs directly inhibit T-cell activation by displaying a repertoire of immune checkpoint ligands, often independently of tumor cell expression (**Figure 6**). PD-L1 on TAMs is a well-documented resistance factor and also confounds tumor proportion score assessment (71, 72). Its regulation is multilayered: TAM-derived PGE2 (71), M2-TAM-secreted TGF-β (73), and apoptotic cell signaling through Mertk (74) all induce PD-L1 expression; the lncRNA ZNF649-AS1 further promotes TAM-dependent PD-L1 (75). A cis-interaction with CD80 can inhibit PD-L1 function (76), while anti-CD40 therapy paradoxically induces IFN-γ-dependent PD-L1 on TAMs, causing acquired resistance (77). CMTM6 and CMTM4 stabilize surface PD-L1 by preventing lysosomal degradation (78); in colorectal cancer, CMTM6 expression in M2 macrophages has been reported in a specific cohort to provide predictive information beyond dMMR status, but requires independent validation (79). Tumor-secreted exosomal CMTM6 also promotes M2 polarization via ERK1/2 (78). Therefore, checkpoint biomarkers should account for cellular source and spatial proximity, not merely total ligand abundance.

Beyond PD-L1, alternative checkpoint-related molecules broaden the myeloid resistance layer. VISTA, Siglec-15, TIM-3-associated programs, IDO-1+ TAM states, and circulating IL1β+ monocytes have each been linked to immune escape or treatment failure in defined settings (71, 77, 80–84). Tumor-intrinsic mechanisms such as HPV E5-mediated MHC downregulation and suppressive cargo delivered by M2-derived extracellular vesicles may converge with these TAM programs to further reinforce resistance (71, 83, 110). This spatially informed interpretation connects checkpoint ligand expression to the broader architecture of TAM-mediated immune exclusion.

Taken together, these findings place TAMs within a myeloid checkpoint layer that can act alongside, but not independently from, tumor-cell checkpoint expression. This layer is shaped by macrophage-intrinsic programs and local TME signals, including apoptotic cell sensing, therapy-induced interferon responses, and post-translational stabilization of PD-L1 (71, 74, 77, 78). The involvement of Siglec-15, TIM-3-related states, checkpoint-dependent TAM activity, and broader innate immune rewiring further indicates that TAMs can restrain antitumor immunity through mechanisms that extend beyond tumor-cell PD-L1 (80–82, 84). TAM-directed intervention may therefore help convert selected “cold” or immune-excluded tumors into more ICB-responsive states, particularly when macrophage-centered checkpoint activity drives T-cell exclusion, defective antigen presentation, suppressive cytokine signaling, or metabolic restraint. This effect is unlikely, however, if poor antigenicity, inadequate T-cell priming, limited vascular access, or stromal exclusion remains dominant. A network-based strategy is therefore more appropriate than targeting isolated checkpoint molecules alone, because checkpoint ligands, phagocytosis checkpoints, and metabolic checkpoints may compensate within the same suppressive niche. These mechanisms may shape both primary and acquired resistance: pre-existing TAM-rich, checkpoint-expressing, immune-excluding niches can blunt initial ICB response, whereas therapy-induced interferon signaling and extracellular vesicle–mediated PD-L1 transfer may sustain resistance under treatment pressure (69, 77).

### 3.4 Metabolic crosstalk and TAM metabolic phenotypes in resistance

**Figure 7:**
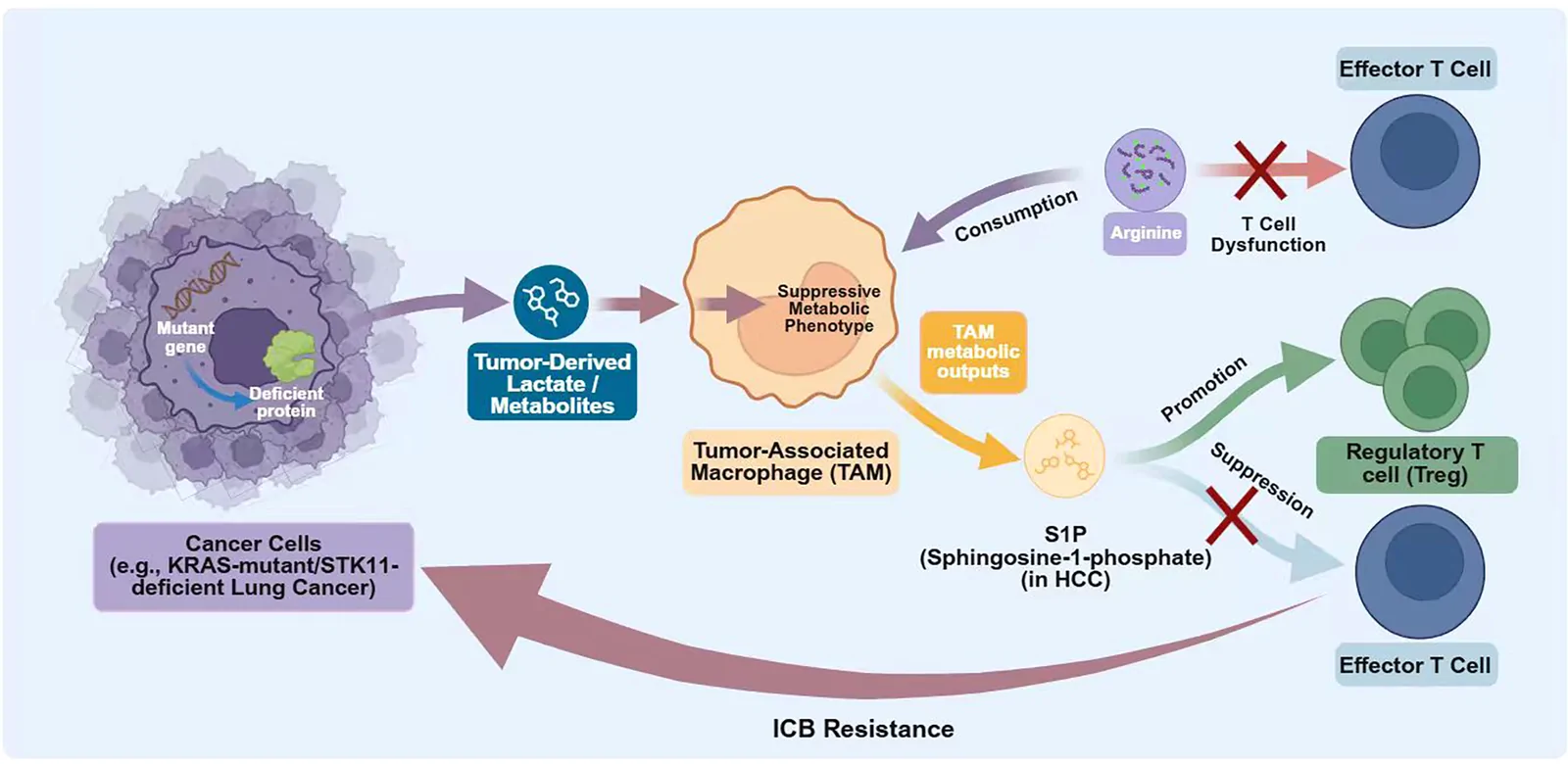
Metabolic crosstalk driving TAM-mediated ICB resistance. Tumor-derived lactate and related metabolites promote suppressive TAM metabolic states. TAM-mediated arginine depletion impairs effector T-cell function, whereas TAM-derived sphingosine-1-phosphate promotes regulatory T cells and suppresses effector T cells, reinforcing ICB resistance.

Metabolic regulation is emerging as a central organizing principle of TAM-mediated resistance, because it simultaneously reprograms macrophage phenotype and compromises T-cell fitness (**Figure 7**). TAM metabolism is both responsive and instructive within the TME. Tumor- and stromal-derived metabolites can educate macrophage state, but macrophages also acquire metabolic phenotypes that reshape neighboring immune, stromal, vascular, and tumor-cell compartments. Recent work has linked macrophage function to glycolytic remodeling, oxidative phosphorylation, fatty acid oxidation, lipid handling, mitochondrial remodeling, amino-acid metabolism, and nutrient-sensing pathways (100). Glycolytic and lactate-conditioned TAM states may further stabilize suppressive transcriptional programs through lactate-dependent histone lactylation, connecting macrophage metabolism with durable immunoregulatory gene expression (101, 102). Other TAM metabolic outputs, including lipid handling, arginine catabolism, adenosine generation, kynurenine–AHR signaling, and UDP/P2Y6-related pathways, can influence T-cell fitness, Treg skewing, antigen presentation, stromal activation, endothelial remodeling, and tumor-cell survival (85–89). These findings support a bidirectional model in which metabolism shapes TAM state and TAMs, in turn, metabolically remodel the TME. Because these macrophage-intrinsic metabolic phenotypes are likely to vary across tumor type, niche, and treatment context, they are not exhaustively reviewed here, but they should be integrated into future analyses of TAM-mediated ICB resistance. Lactate illustrates this convergence: in LKB1-deficient lung adenocarcinoma, tumor-derived lactate drives M2 polarization and T-cell hypofunction, and blocking lactate transport can disrupt this metabolic axis of primary resistance (18). In PDAC, lactate triggers ENSA lactylation in tumor cells, fueling a STAT3/CCL2 feed-forward loop that amplifies macrophage recruitment (6). In HCC, TAM-derived sphingosine-1-phosphate (S1P) promotes M2 polarization and Treg expansion while suppressing effector T cells (85). Tryptophan catabolism generates L-kynurenine, which activates the AHR pathway to expand Tregs and M2-like TAMs (86). Extracellular adenosine signals through the A2a receptor on TAMs to deepen immunosuppression (87). In NSCLC, the P2X7/STAT6 axis drives M2-like polarization and CD8+ T-cell exhaustion (10); concurrent elevation of cancer cell cytidine deaminase increases extracellular UDP, which engages P2Y6 receptors on TAMs to restrict T-cell infiltration (88). Nutrient competition also plays a role: TAMs deplete L-arginine via arginase 1, starving T cells of a metabolite critical for proliferation (89). These findings support a model in which metabolic pathways organize both macrophage identity and T-cell dysfunction, making metabolism a structural driver rather than an auxiliary feature of ICB resistance.

An *in vivo* CRISPR screen in KRAS-mutant lung adenocarcinoma identified the histone chaperone Asf1a as a regulator at this metabolic-immune interface; its loss upregulates GM-CSF, shifting macrophages toward inflammatory antitumor programs and restoring anti-PD-1 sensitivity (22). In prostate cancer, a TAM subset accumulates lipids via the MARCO scavenger receptor (19). While these pathways are well validated preclinically, the metabolic plasticity of the TME likely enables compensation when single nodes are blocked, indicating that combined or upstream targeting may be required for durable efficacy.

Interactions with the stromal compartment further strengthen TAM-associated metabolic suppression and can generate therapy-specific resistance programs. In bladder cancer, inflammatory CAF-based subtypes have been linked to prognosis and ICB response (51). Similarly, in mismatch repair-deficient colorectal cancer, a TGF-β-dependent stromal program identifies an ICB-resistant subgroup characterized by enrichment of M2-polarized macrophages (111). These findings suggest that stromal signals not only shape macrophage phenotype, but also influence whether tumors remain responsive to ICB. Non-macrophage immune compartments can contribute to the same resistance ecology. For example, IL-17A-producing γδ T cells promote resistance to CDK4/6 inhibition in hormone receptor-positive breast cancer through CX3CR1+ macrophages (112). In another setting, HLA-G–KIR2DL4 interactions between tumor cells and NK cells suppress IFN-γ production and contribute to trastuzumab resistance (113). Although this pathway is not macrophage-intrinsic, it illustrates how non-myeloid immune circuits may intersect with TAM-centered stromal–immune networks. Exosome-related risk signatures in TNBC have also been associated with enrichment of M2-like macrophages, suggesting that extracellular vesicle programs may reflect, or contribute to, broader myeloid remodeling in the TME (114). CAFs are central components of this network because they release factors that regulate TAM metabolism, differentiation, and suppressive function. In gastric cancer, CAF-derived C3 promotes the differentiation of SPP1+ TAMs (25). In bladder cancer, serum amyloid A recruits immunosuppressive macrophages (90). In pancreatic cancer, CAF-derived TSG-6 engages CD44 on TAMs and induces an Arg1-high phenotype (5). This communication is bidirectional. IL-15Rα+ TAMs reduce CX3CL1 expression in tumor cells, thereby limiting CD8+ T-cell infiltration (115), whereas tumor-derived SPP1 promotes an immunosuppressive APOE+ TAM subset in ccRCC (116). In TNBC, CA9+ CAFs interact with SPP1+ TAMs through the VEGFA/NRP2 axis (30). Beyond soluble mediators, stromal organization can also impose physical barriers to immunity. In HCC, M2BP-driven macrophage-coated tumor clusters generate local immune-deprived zones that restrict T-cell entry (40). Together, these observations place TAMs within a broader stromal–immune resistance network. They also suggest that metabolic therapies are likely to be more effective when combined with approaches that dismantle the stromal architecture sustaining macrophage-mediated immune exclusion.

### 3.5 Impairment of phagocytosis and antigen presentation

**Figure 8:**
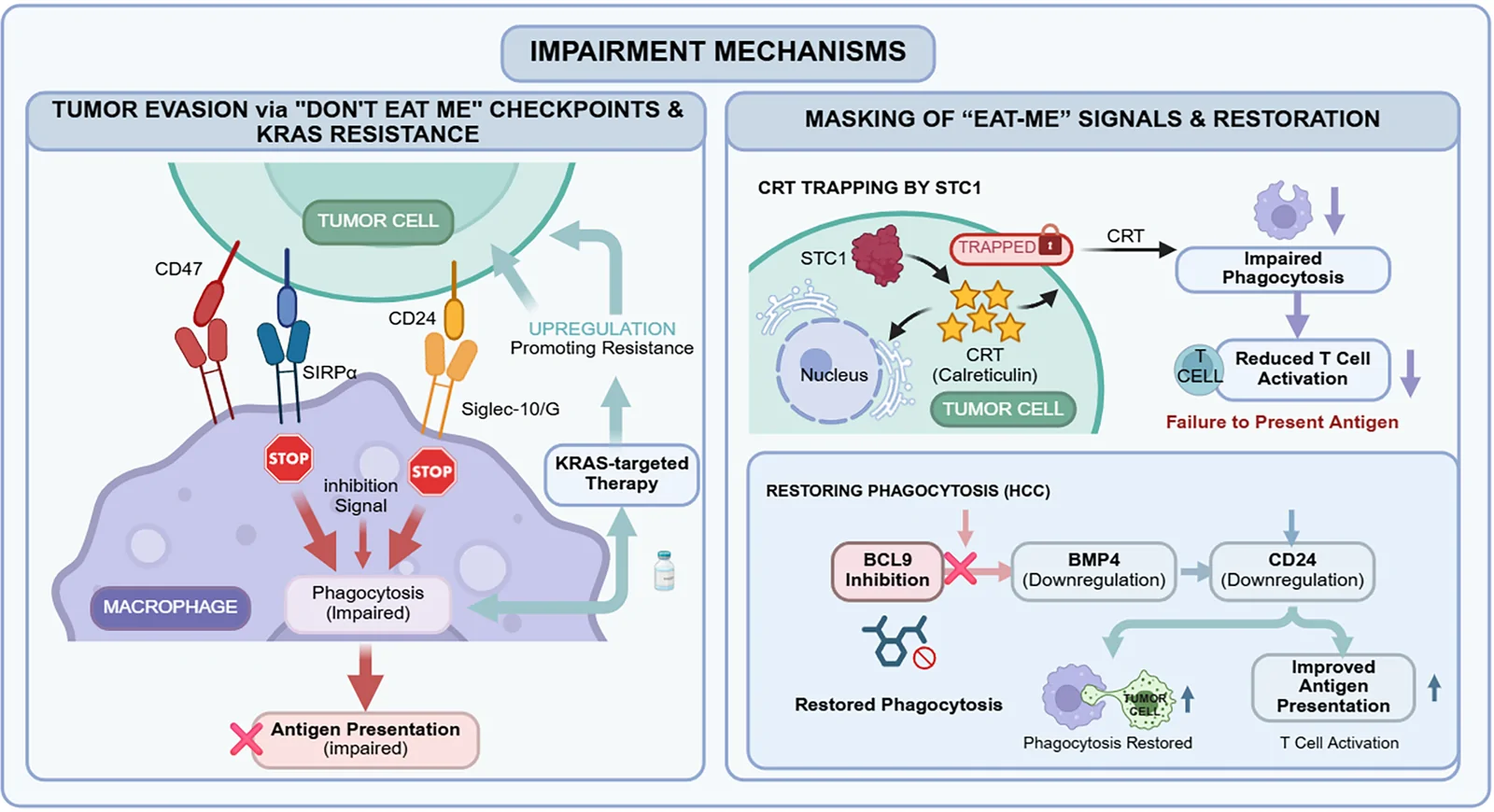
Impaired and restored macrophage phagocytosis and antigen presentation. CD47–SIRPα and CD24–Siglec-10/G checkpoints, KRAS therapy-associated upregulation of antiphagocytic signals, and STC1-mediated calreticulin trapping impair phagocytosis, antigen presentation, and T-cell activation. In hepatocellular carcinoma, BCL9 inhibition downregulates BMP4 and CD24, restoring phagocytosis and antigen presentation.

Tumor cells can avoid macrophage-mediated clearance by activating “don’t eat me” pathways, among which CD47–SIRPα and CD24–Siglec-10/G are the best characterized (91, 92) (**Figure 8**). The importance of these checkpoints varies across tumor contexts and may become more pronounced during treatment. For example, KRAS-targeted therapy can unexpectedly increase the expression of anti-phagocytic signals, thereby contributing to adaptive resistance (93). Tumors may also suppress phagocytosis by limiting the availability of pro-phagocytic cues. STC1, for instance, retains calreticulin within tumor cells, which reduces macrophage engulfment and weakens subsequent T-cell activation (94). In HCC, inhibition of BCL9 decreases BMP4 secretion, leading to reduced CD24 expression and restoration of macrophage phagocytic activity (95). Thus, defective phagocytosis should not be viewed solely as an innate immune escape mechanism; it also represents a link between failed macrophage clearance and insufficient activation of adaptive antitumor immunity.

### 3.6 Direct interference with therapeutic antibodies

**Figure 9:**
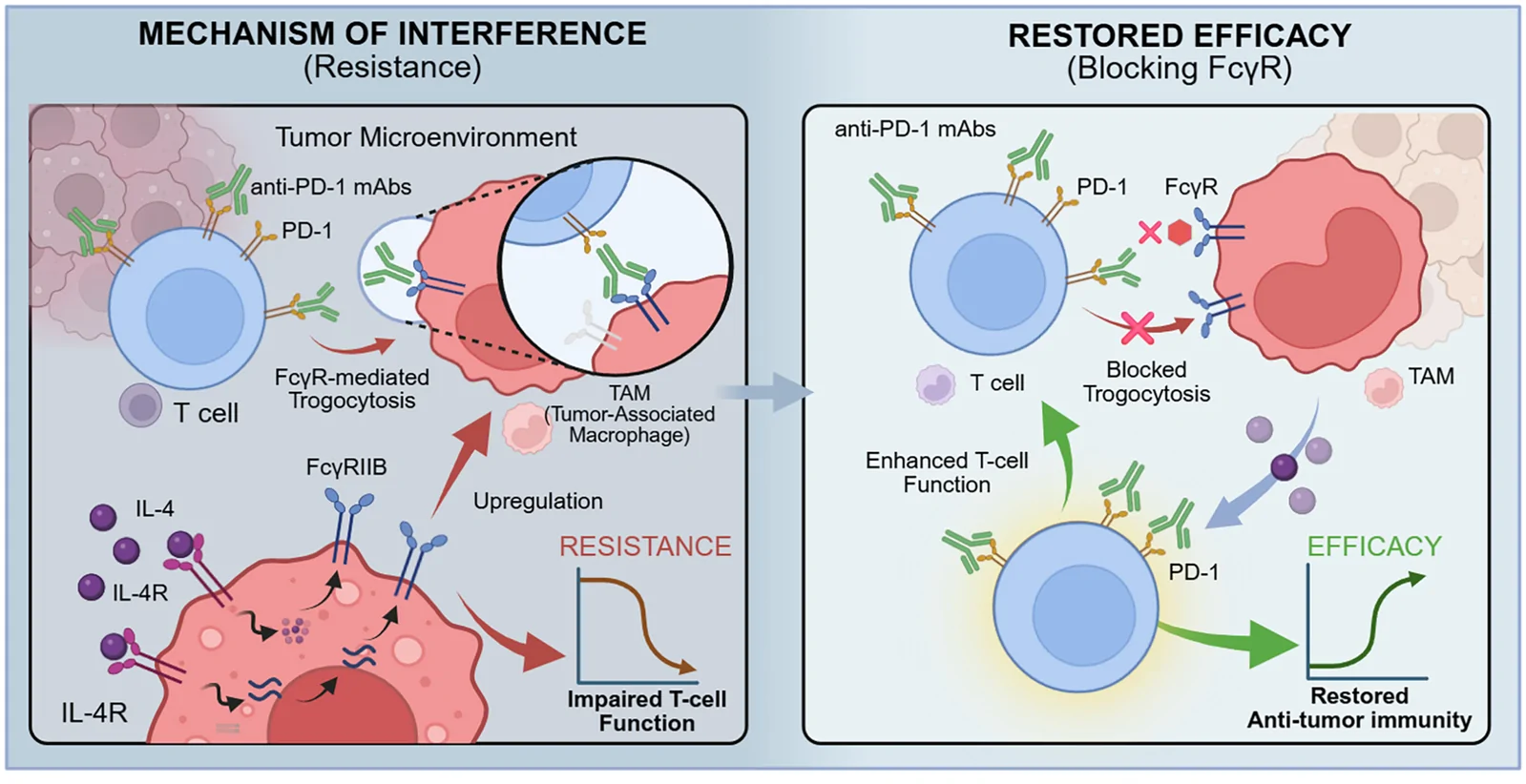
Fcγ receptor-mediated interference with anti-PD-1 antibodies. TAMs remove anti-PD-1 antibodies from T cells through Fcγ receptor-mediated trogocytosis. IL-4/IL-4R signaling upregulates FcγRIIB and promotes resistance, whereas Fcγ receptor blockade prevents trogocytosis and restores T-cell function and antitumor immunity.

TAMs can directly subvert ICB by stripping anti-PD-1 antibodies from T cells via FcγR-mediated trogocytosis; blocking this interaction restores efficacy (96) (**Figure 9**). IL-4/IL-4R signaling potentiates this mechanism by upregulating inhibitory FcγRIIB on M2 macrophages (52). This reframes TAMs not merely as immune suppressors but as pharmacologic sinks. Importantly, the ICB–myeloid interaction may be bidirectional in some contexts. PD-1 blockade has been reported to affect PD-1-expressing TAMs directly, promoting a shift from a suppressive pro-tumoral state toward a more tumoricidal macrophage phenotype and restoring phagocytic activity against cancer cells (117). This bidirectionality underscores why TAM-directed therapy should preserve or restore macrophage effector functions rather than indiscriminately eliminating the entire macrophage compartment.

Across the mechanisms summarized in **Table 2**, **Supplementary Table 2**, and **Figures 3**-**9**, TAMs emerge as active drivers of local immune failure rather than passive markers of an inflamed or suppressive tumor microenvironment. They mediate resistance through macrophage education, soluble mediator circuits, checkpoint ligand expression, metabolic–stromal coupling, impaired phagocytosis, and Fc receptor–dependent antibody handling. Although the upstream cues differ among tumors and can evolve during treatment, the downstream consequences are recurrent: weakened effector immunity, checkpoint-mediated suppression, defective phagocytosis and antigen presentation, interference with therapeutic antibodies, and spatial immune exclusion within macrophage–stromal–tumor cell neighborhoods. In many tumors, these processes coexist and reinforce one another, producing a layered barrier to effective ICB. Determining which TAM program is dominant in a specific context is therefore central to mechanism-matched TAM-directed therapy and provides the basis for the therapeutic strategies discussed below.

## 4 TAM-directed therapeutic strategies to overcome ICB resistance

**Figure 10:**
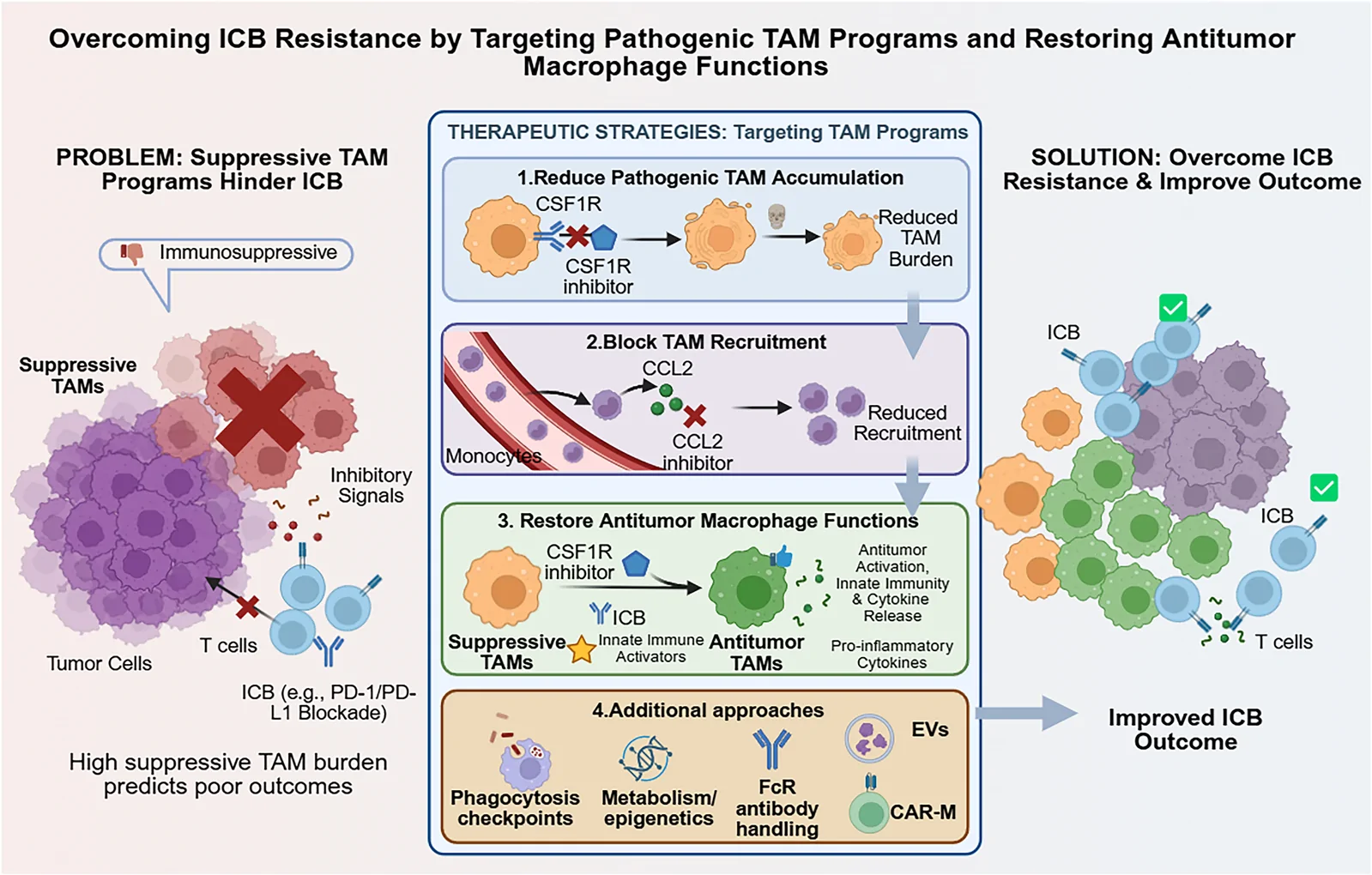
TAM-directed strategies to overcome ICB resistance. TAM-directed therapy aims to reduce pathogenic macrophage accumulation, block recruitment, interrupt tumor-promoting TAM programs, and restore antitumor macrophage functions. The therapeutic goal is selective remodeling of resistance-driving TAM states rather than indiscriminate macrophage elimination.

The final stage of this framework focuses on therapeutic interception—how to disrupt macrophage recruitment, education, or suppressive activity while preserving beneficial macrophage functions. Given their central role in ICB resistance, TAMs have become important therapeutic targets for restoring antitumor immunity (**Figure 10**). These strategies are better understood as therapeutic entry points than as a fixed classification. They include limiting immunosuppressive macrophage accumulation, blocking monocyte or macrophage recruitment, and reprogramming pathogenic TAM states toward antitumor effector functions, including phagocytosis, antigen presentation, inflammatory activation, and tumoricidal activity. More recent approaches extend beyond polarization-based reprogramming. They include selective targeting of tumor-promoting TAM programs, preservation or restoration of antitumor macrophage functions, phagocytosis-checkpoint enhancement, modulation of Fc receptor–dependent antibody handling, metabolic and epigenetic targeting, spatially selective delivery, extracellular vesicle-based strategies, and macrophage-based cellular therapies (118, 119). For example, CSF1R inhibition has been used to deplete or remodel TAM populations, particularly in tumors where high macrophage infiltration is associated with poor outcome (120). Recruitment blockade, especially through chemokine axes such as CCL2–CCR2, aims to limit the continuous replenishment of suppressive macrophages within the TME (121, 122). In parallel, macrophage reprogramming strategies attempt to restore defined macrophage effector functions, including inflammatory activation, tumoricidal activity, phagocytosis, antigen presentation, and macrophage–T-cell cooperation, rather than simply reduce TAM numbers. CSF1R inhibition may contribute to this phenotypic shift (121), while ICB and innate immune activators can promote immune metabolism, cytokine release, and macrophage-associated antitumor responses (122). Nanoparticle-based platforms further provide a way to deliver TAM-modulating agents more selectively and may enhance the efficacy of ICB while reducing systemic exposure (121). **Figure 10** summarizes the therapeutic logic of TAM-directed therapy in ICB resistance. **Table 3** further classifies major TAM-directed strategies according to their mechanism of action, representative approaches, development stage, and translational considerations. Representative examples are provided in **Supplementary Table 3**.

**Table 3:** TAM-directed therapeutic strategies classified by mechanism of action and developmental stage.

Therapeutic logic	Representative targets or approaches	Dominant mechanism of action	Developmental stage	Key translational consideration	Ref.
TAM depletion/remodeling	CSF1/CSF1R inhibition	Reduces or remodels CSF1R-dependent macrophage populations	Preclinical and clinical evaluation	Variable clinical benefit; resistant TAM subsets and loss of beneficial macrophage functions remain concerns	(120, 121)
Recruitment blockade	CCL2–CCR2, CCR2/5, CCR1	Limits monocyte recruitment and TAM replenishment	Preclinical and clinical evaluation	Chemokine redundancy and rebound monocyte influx may limit efficacy	(120, 121, 123)
Functional reprogramming	PI3Kγ/PI3Kγδ, Akt/mTOR-related signaling, TYRO3, STAT3/NF-κB/PARP14-related programs	Redirects suppressive TAMs toward less immunosuppressive or more inflammatory states	Mainly preclinical, with selected clinical evaluation	Requires tumor-context selection, functional monitoring, and rational combination with ICB	(61, 123–134)
Metabolic/epigenetic reprogramming	Lactate-related pathways, 2-DG, APOC1 inhibition, methionine restriction, lncRNA/m6A-related modulation	Reverses metabolism- or epigenetics-driven suppressive TAM states	Mainly preclinical, with selected clinically available agents	Should be guided by dominant metabolism-dependent TAM programs rather than total TAM abundance	(60, 100–102, 135–140)
Other TAM-modulating and delivery-based approaches	Repurposed agents, oncolytic viruses, STING agonist delivery, nanoparticle/EV-based platforms	Remodels TAM states or improves local delivery of TAM-modulating agents	Mainly preclinical, with selected early clinical evaluation	Mechanistic heterogeneity requires target prioritization, delivery specificity, and biomarker-guided selection	(39, 83, 121, 140–147)
Phagocytosis enhancement	CD47–SIRPα blockade, CD24–Siglec-10/G targeting, calreticulin restoration	Restores macrophage-mediated tumor cell engulfment and may improve antigen presentation	Preclinical and clinical evaluation for CD47–SIRPα; mainly preclinical for other axes	Hematologic toxicity, tumor-context specificity, and adaptive “don’t eat me” pathways remain barriers	(91–95, 148)
Tumor–TAM circuit disruption	CCL15–CCR1, CCL3–CCR1, GPSM1–MEIS3–CSF1, circRNA- or integrin-related tumor–myeloid circuits	Blocks reciprocal tumor–macrophage loops that maintain suppressive TAM states	Mainly preclinical	Requires identification of the dominant tumor–myeloid circuit in each tumor context	(31, 56, 123, 127)

However, the key clinical issue is not only whether TAMs can be depleted, but whether the specific macrophage program responsible for resistance can be identified and safely interrupted. Macrophages also support antigen presentation, phagocytosis, tissue repair, and antibody-dependent immune responses. Therefore, indiscriminate elimination of the macrophage compartment may weaken, rather than strengthen, antitumor immunity in some settings. A more rational therapeutic goal is to suppress the pathogenic TAM states that maintain immune resistance while preserving, or restoring, macrophage functions that contribute to tumor clearance. This need for selectivity provides the basis for the pathway-, metabolism-, phagocytosis-, and delivery-focused strategies discussed below.

### 4.1 Targeting key signaling pathways in TAMs

Dysregulated intracellular signaling pathways in TAMs maintain immunosuppressive macrophage states and contribute to ICB resistance. However, the therapeutic relevance of these pathways should not be judged simply by whether they correlate with a suppressive polarization marker profile. A more useful interpretation is to ask whether a pathway functions as a dominant education signal, a maintenance circuit, or a compensatory escape route after therapy. The PI3K/AKT/mTOR axis illustrates this distinction: TYRO3-associated resistance, Akt-dependent accumulation of M2-like TAMs after radiotherapy plus checkpoint blockade, and PI3Kγ or PI3Kγδ inhibition in glioma and radiotherapy-combination settings all point to a shared principle that survival and metabolic signaling can stabilize suppressive macrophage states (61, 124–126). TAM signaling is therefore best targeted by identifying the macrophage program that sustains resistance in a given therapeutic context, rather than by applying pathway inhibition indiscriminately.

Crosstalk between cancer stem cells (CSCs) and TAMs drives resistance. In bladder cancer, CSCs use the CCL15-CCR1 axis to polarize macrophages to an SPP1+ phenotype, causing CD8+ T-cell exclusion and ICB resistance (127). In glioblastoma (GBM), SPP1+ TAMs also drive anti-PD-1 resistance; a tumor-derived circRNA (circSDHAF2) induces this phenotype via exosomal transfer of stabilized ITGA5, and blocking ITGA5 enhances anti-PD-1 therapy (31). CCR1 inhibition may overcome ICB resistance by disrupting CCR1+ macrophage-associated suppressive niches and restoring CD8+ T-cell accessibility or function (123). Therefore, successful pathway targeting will likely require disrupting the cellular circuit that maintains TAM function, rather than inhibiting a single macrophage marker in isolation.

Acidosis within the TME promotes an immunosuppressive TAM phenotype via the proton-sensing receptor GPR65. Pharmacologic GPR65 inhibition reverses this effect and synergizes with anti-PD-1 therapy (58). In melanoma, HPGDS expression in TAMs signifies anti-PD-1 resistance by producing PGD2, which maintains a protumoral state and inhibits CD8+ T cells. HPGDS inhibition reprograms TAMs and sensitizes tumors to anti-PD-1 blockade (128). In colorectal cancer, elevated tumor cell GPSM1 drives M2 polarization and anti-PD-1 resistance via the GPSM1-MEIS3-CSF1 axis; targeting this pathway enhances anti-PD-1 efficacy (56). Although these pathways differ molecularly, they converge on the same therapeutic question: whether blocking the upstream stress-sensing cue can durably reprogram macrophages once the broader TME remains hypoxic, acidic, and metabolically hostile. This convergence suggests that stress-response pathways may be most effective as combination targets paired with metabolic or stromal remodeling strategies.

The NF-κB and STAT3 pathways are also pivotal. The STAT3 inhibitor ACT001 reduces tumor PD-L1 and M2 markers in glioma (129). NF-κB activation in TAMs drives drug resistance in pancreatic cancer (130) and chemoresistance in osteosarcoma (131). Conversely, cIAP1/2 antagonists activate non-canonical NF-κB, stimulating a T-cell–macrophage axis that makes TAMs tumoricidal (43). Targeting PARP14 with anti-PD-1 therapy reduces suppressive TAM programs and expands proinflammatory macrophage states to overcome ICB resistance (132). Other targets include the TNF/p65 axis in GBM (133) and p38 MAPK, whose inhibition reduces glioma-associated macrophages and enhances anti-PD-L1 efficacy (134). The antidepressant imipramine also reprograms TAMs by inhibiting histamine receptor signaling (42). This duality cautions against labeling pathways as simply “pro-tumor” or “anti-tumor” without considering cell type, timing, and signaling branch. Accordingly, pathway-directed TAM therapy requires functional monitoring, because the same signaling family may either sustain suppression or enable macrophage-mediated tumor clearance depending on context.

Targeting tumor-intrinsic pathways can secondarily reprogram TAMs. In primary central nervous system lymphoma, combining XPO1 and BTK inhibitors shifts macrophages toward an inflammatory antitumor phenotype (149). Activating the STING pathway in TAMs can overcome hormone therapy resistance. In prostate cancer, targeted delivery of a STING agonist to protumoral FR-β+ (folate receptor beta-positive) TAMs induces IFN-β production, activates cytotoxic lymphocytes, and delays resistance (39). In breast cancer, the lncRNA NEAT1 promotes paclitaxel resistance and M2 polarization by sponging miR-133b to upregulate PD-L1; silencing NEAT1 reverses both effects (150). These findings broaden the therapeutic concept from “target TAMs directly” to “target the tumor–macrophage circuit.” This circuit-based view connects intracellular signaling interventions to the broader principle that TAM resistance is often sustained by reciprocal tumor–myeloid communication.

Targeting the CD47-SIRPα “don’t eat me” checkpoint unleashes TAM phagocytosis, showing potent activity in lymphoma (91). Therapeutic pressure often paradoxically fuels adaptive resistance. For example, KRAS inhibition can inadvertently trigger the upregulation of the “don’t eat me” signals CD47 and CD24. These findings suggest that combined targeting of KRAS signaling, phagocytosis checkpoints, and PD-L1 may be required to prevent adaptive immune escape in this setting (93). Environmental stressors within the TME may also shape therapeutic resistance. In hypoxic niches of hepatocellular carcinoma, TREM-1 induction on TAMs promotes regulatory T-cell recruitment and contributes to resistance to anti-PD-L1 therapy; targeting TREM-1 may reverse this barrier (35). Distinct metabolic programs further drive evasion; in gastric cancer, CLEVER-1+ TAMs exploit a PPARγ-dependent axis to enforce immunosuppression. Bexmarilimab-mediated CLEVER-1 blockade may reprogram TAMs toward a more inflammatory state and enhance their cooperation with anti-PD-1 treatment (151). These examples support a therapeutic logic in which TAM-directed agents should be selected according to the dominant resistance layer—phagocytic blockade, hypoxic niche protection, or metabolic immune suppression.

**Figure 11:**
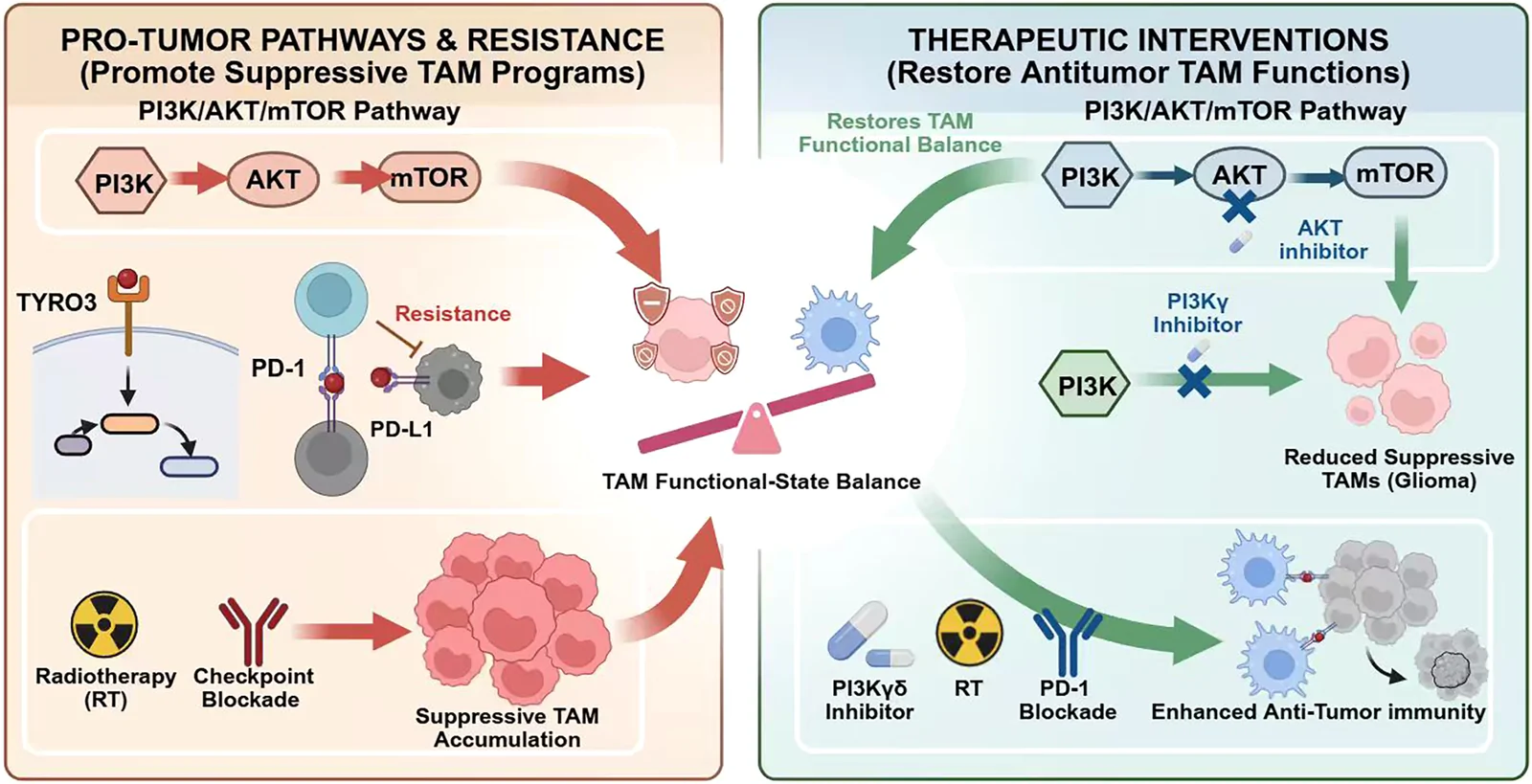
Targeting PI3K/AKT/mTOR signaling in TAMs. The figure contrasts PI3K/AKT/mTOR-associated protumor signaling and suppressive TAM accumulation with therapeutic inhibition of AKT, PI3Kγ, or PI3Kγδ. These interventions restore TAM functional balance, reduce suppressive TAMs, and enhance antitumor immunity and PD-1 blockade efficacy.

Furthermore, the redundancy of survival signaling remains a pervasive challenge, as tumor-derived IL-34 can engage CSF1R to sustain pathogenic M2-like polarization, underpinning broad resistance to regimens ranging from ICB and PARP inhibitors to radiotherapy. Targeting this axis can overcome resistance (66). In NSCLC, combining an EGFR inhibitor with an anti-angiogenic agent reverses resistance by downregulating protumoral TAMs (152). Similarly, in melanoma, combining a BRAF inhibitor with anti-VEGFA promotes infiltration of antitumor macrophage populations in a GM-CSF-dependent manner, sensitizing tumors to anti-PD-1 therapy (153). In breast cancer, PARP inhibitor resistance is associated with a TAM subpopulation with Rps19/C5aR1 signaling; C5aR1 inhibition overcomes this by selectively depleting pro-tumoral macrophages (36). The key translational implication is that TAM biology should be incorporated into the design of targeted-therapy and ICB combinations from the outset, rather than treated as a downstream biomarker of resistance. Together, the pathways summarized in **Figure 11** argue for program-matched pathway inhibition guided by macrophage state, tumor genotype, and treatment-induced myeloid adaptation.

### 4.2 Modulating TAM metabolism and epigenetics

Emerging evidence suggests that metabolic and epigenetic reprogramming of TAMs may offer actionable opportunities to overcome resistance to ICB. Rather than simply eliminating macrophages, these strategies aim to interfere with the metabolic dependencies and regulatory circuits that sustain suppressive TAM states. From this perspective, metabolism- and epigenetics-directed strategies should not be framed as generic “M1 repolarization” approaches. Their more precise goal is to identify and reverse the dominant metabolic-epigenetic program that maintains macrophage-driven resistance in a given tumor, such as lactate-conditioned chromatin remodeling, lipid-associated suppressive programming, amino-acid-dependent macrophage education, or non-coding RNA–associated immune remodeling (60, 100–102). In prostate cancer, dietary modulation such as a ketogenic diet has been reported to reverse resistance to combined anti-PD-1/CTLA-4 therapy by redirecting suppressive macrophage programs toward more inflammatory and immune-supportive states rather than simply restoring a numerical M1/M2 balance (135). In colorectal cancer, inhibition of glucose metabolism with agents such as 2-DG disrupts the CAF–EDEM3 axis, which otherwise stabilizes PD-L1 expression and promotes recruitment of suppressive TAM populations, thereby enhancing the efficacy of anti-PD-1 therapy (136). In HCC, targeting APOC1 induces ferroptosis in TAMs and disrupts lipid-associated suppressive macrophage programs, improving the response to anti-PD-1 treatment (100, 137). Nutrient availability can also shape macrophage function: methionine restriction in gastric cancer suppresses MIF, promotes a more inflammatory macrophage state, and sensitizes tumors to ICB (60), whereas altered carnitine metabolism has been implicated in resistance in NSCLC (138). Epigenetic and post-transcriptional mechanisms add another layer of regulation to these metabolic programs. More broadly, non-coding RNAs and extracellular vesicles can link metabolic adaptation to immune escape. lncRNAs contribute to immunosuppressive remodeling of the tumor immune microenvironment and may participate in therapy resistance (139), while m6A-related lncRNA signatures in glioblastoma have been investigated as potential predictors of ICB response (140). In parallel, tumor-derived exosomal PD-L1 can drive systemic immunosuppression and counteract anti-PD-1 activity (69). Exosome-related gene signatures in TNBC similarly appear to reflect the immune status of the TME and may have prognostic relevance (114). In addition, CMTM6 and CMTM4 connect post-translational stabilization of PD-L1 with broader remodeling of the TME, positioning them as candidate biomarkers and therapeutic modifiers rather than merely PD-L1-regulating molecules (78). Taken together, these findings argue that metabolic and epigenetic features should be incorporated into biomarker development, as they may help explain why transcriptionally similar TAM populations show divergent functional behavior after treatment.

**Figure 12:**
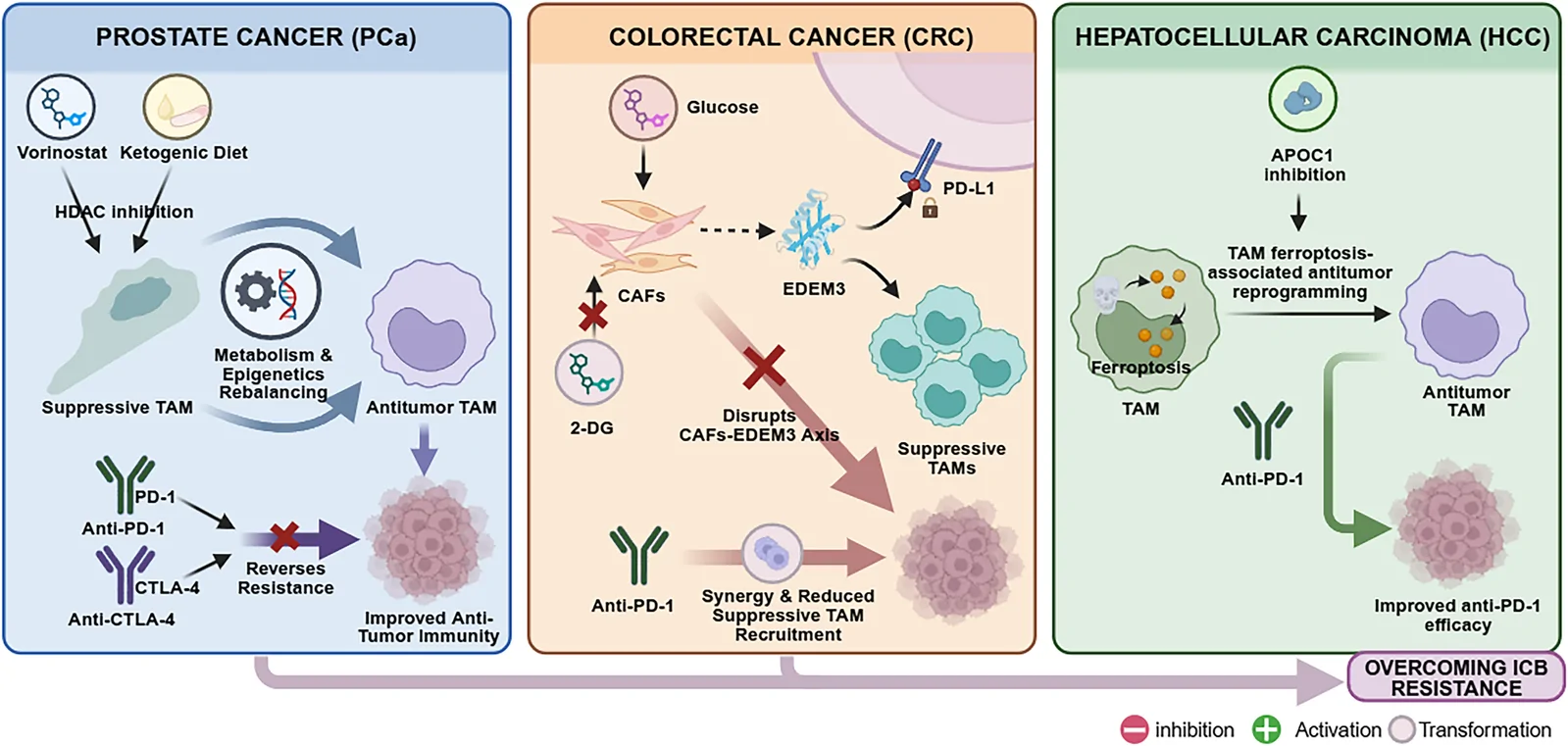
Metabolic and epigenetic reprogramming of TAMs in prostate, colorectal, and hepatocellular cancers. In prostate cancer, vorinostat and a ketogenic diet promote metabolic and epigenetic rebalancing and reverse resistance to PD-1 and CTLA-4 blockade. In colorectal cancer, 2-DG disrupts the CAF–EDEM3 axis and reduces suppressive TAM recruitment. In hepatocellular carcinoma, APOC1 inhibition induces ferroptosis-associated antitumor TAM reprogramming and improves anti-PD-1 efficacy.

Overall, the interventions summarized in **Figure 12** suggest that TAM metabolism and epigenetics are most clinically relevant when they define persistent, reversible, and measurable macrophage states. Their therapeutic promise will depend on identifying which patients harbor metabolism-dependent TAM resistance and whether these states can be reprogrammed without impairing systemic immune or metabolic homeostasis.

### 4.3 Targeting other pathways and repurposed compounds

In addition to pathway-specific interventions, repurposed compounds and alternative targeting strategies have shown potential to modulate TAM function and enhance ICB responses. Although diverse in origin, these approaches converge on a limited number of biological processes that reshape macrophage function and the broader tumor microenvironment. Rather than adding another list of agents, this section identifies shared therapeutic outputs across diverse TAM-modulating strategies.

A substantial proportion of these strategies appear to work, at least in part, by reshaping macrophage functional states and the signaling circuits that maintain an immunosuppressive tumor milieu. In glioblastoma, HSPA7 silencing increases sensitivity to anti–PD-1 treatment, whereas COX-2 inhibition has been reported to attenuate suppressive macrophage programming (140, 141). Comparable macrophage-centered effects have also been described in other tumor contexts. For example, honokiol limits suppressive macrophage polarization programs in NSCLC (142), while PARP14 inhibition in melanoma and the combination of selinexor with ibrutinib in central nervous system lymphoma can shift TAMs toward a less immunosuppressive phenotype (132, 149). In triple-negative breast cancer, Oligo-Fucoidan combined with olaparib, as well as baohuoside I through interference with extracellular vesicle–mediated communication, promotes a more pro-inflammatory macrophage state and enhances the response to cytotoxic therapy (143, 144). Nevertheless, the translational value of these interventions should not be judged solely by their ability to alter macrophage markers in preclinical models. More critical considerations include whether the reprogrammed state is durable, whether the intervention selectively targets pathogenic TAM populations without broadly disrupting protective myeloid functions, and whether it can be integrated safely and effectively with established immune checkpoint blockade regimens.

In addition to approaches that act directly on macrophages, several therapeutic strategies highlight the need to remodel the tumor microenvironment more broadly. In NSCLC, anlotinib combined with PD-1 blockade produces detectable changes in the immune landscape (107), suggesting that anti-angiogenic and immunotherapeutic pressures may jointly influence myeloid cell behavior. Other repurposed agents may act through distinct but convergent mechanisms; for instance, rimantadine has been shown to overcome HPV16 E5–mediated resistance to checkpoint inhibition (83). Oncolytic viruses represent another complementary modality. Newcastle disease virus, for example, can induce a more inflammatory microenvironment, thereby creating conditions that may favor improved responses to immune checkpoint blockade (145). Although these interventions differ substantially in their primary targets and mechanisms of action, they converge on a common principle: macrophage phenotypes are not fixed, but are shaped by the broader immune and stromal context generated by therapy. This observation supports a shift in the design of TAM-directed treatments, from strategies focused mainly on macrophage elimination toward approaches that deliberately guide tumor microenvironment state transitions.

More recent efforts have focused on engineering strategies that integrate delivery and immune modulation. A biomimetic “nano decoy” has been reported to reverse post–radiofrequency ablation–associated immunosuppression by inducing immunogenic cell death and restoring antitumor immune activity (146). These platforms may be particularly useful when the therapeutic goal is to alter macrophage function without eliminating tissue-resident macrophages in healthy organs. Thus, delivery technology is central to making TAM reprogramming clinically feasible.

**Figure 13:**
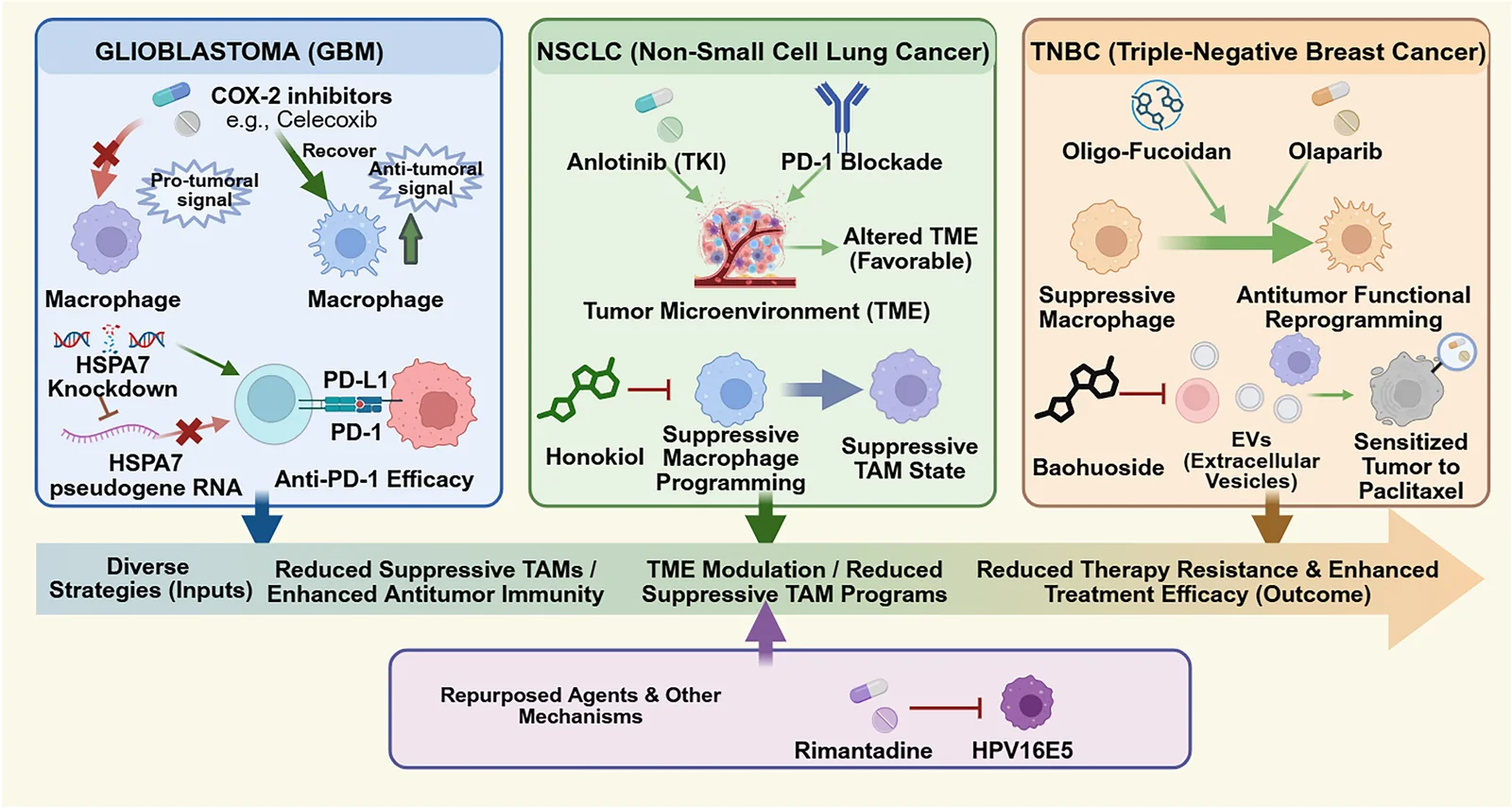
Repurposed and alternative TAM-modulating strategies. In glioblastoma, COX-2 inhibition suppresses protumoral signaling, while HSPA7 pseudogene RNA knockdown enhances anti-PD-1 efficacy. In non-small cell lung cancer, anlotinib plus PD-1 blockade remodels the tumor microenvironment, and honokiol inhibits suppressive macrophage programming. In triple-negative breast cancer, oligo-fucoidan plus olaparib promotes antitumor macrophage reprogramming, while baohuoside I blocks extracellular vesicle-mediated signaling and sensitizes tumors to paclitaxel. Rimantadine inhibits HPV16 E5-mediated resistance.

Taken together, the repurposed and alternative approaches summarized in **Figure 13** indicate that mechanistically diverse interventions can converge on a shared set of TAM-centered outputs. The major challenge is to distinguish interventions that transiently perturb macrophage markers from those that durably reset the tumor immune ecosystem. This distinction provides a bridge to the clinical translation section, where biomarker selection, toxicity, trial design, and spatial monitoring determine whether TAM-modulating strategies can succeed in patients. Representative pathway-level, metabolic, epigenetic, phagocytosis-directed, repurposed, and delivery-based TAM-directed interventions are summarized in **Supplementary Table 3**.

## 5 Clinical translation and future perspectives

**Figure 14:**
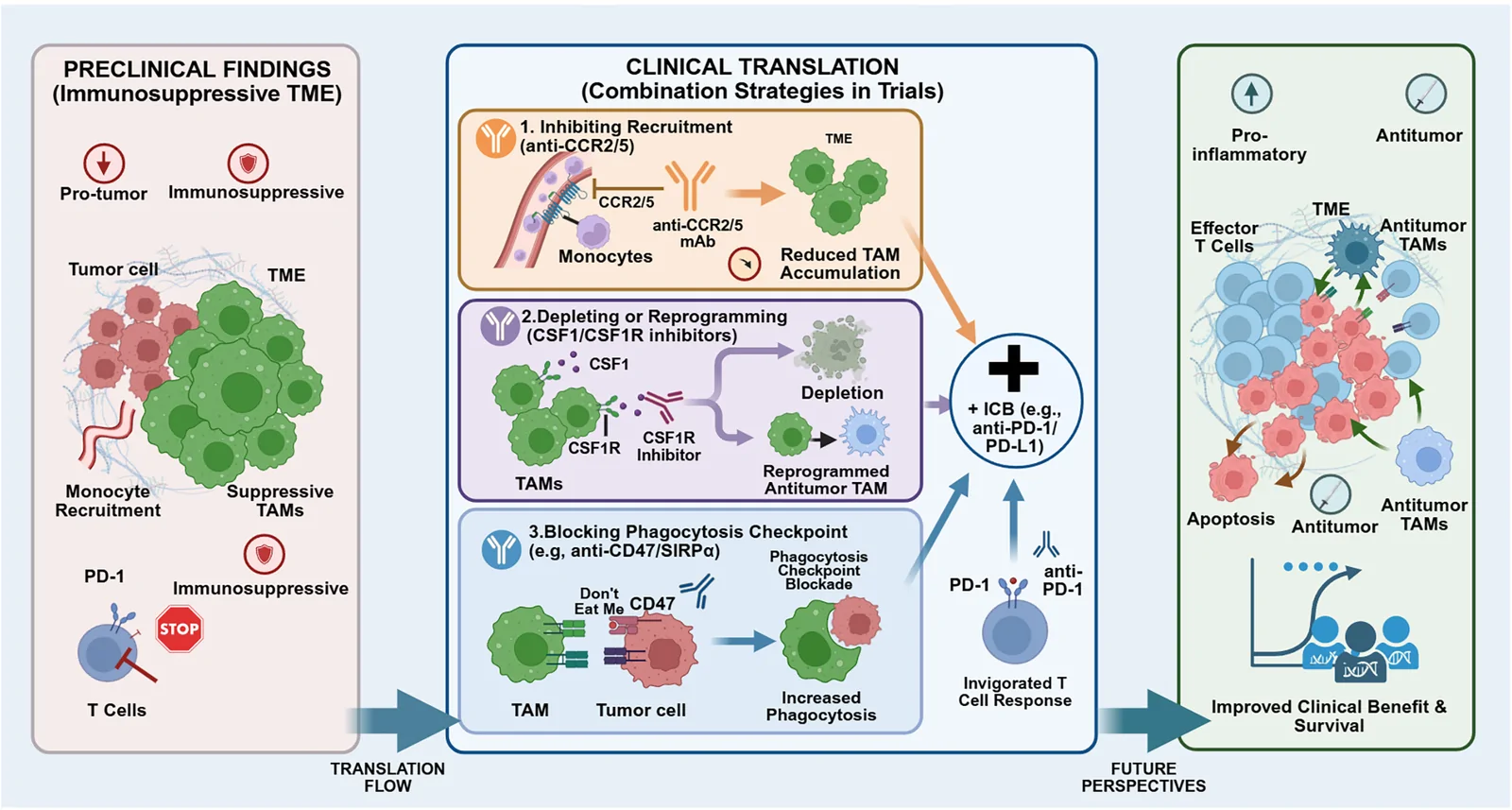
Clinical translation of TAM-directed therapy. The figure links an immunosuppressive preclinical tumor microenvironment to clinical combination strategies that inhibit monocyte recruitment, deplete or reprogram TAMs through CSF1/CSF1R blockade, and block phagocytosis checkpoints. Combined with ICB, these approaches aim to restore effector T-cell activity, promote antitumor TAM states and tumor-cell apoptosis, and improve clinical benefit and survival.

The clinical translation of TAM-directed therapy can be viewed as the unresolved end point of the staged framework. Even when macrophage recruitment, education, or suppressive execution is well defined in models, clinical benefit depends on identifying the dominant resistance program, targeting it safely, and monitoring it with robust biomarkers (**Figure 14**). Preclinical advances have accelerated clinical testing of TAM-directed strategies, shifting TAMs from descriptive biomarkers to active therapeutic targets in ICB combination regimens. Current TAM-directed strategies include chemokine receptor blockade to limit monocyte recruitment, CSF1/CSF1R inhibition to interfere with macrophage survival signals, and blockade of “don’t eat me” checkpoints. The latter concept is supported by early clinical activity of anti-CD47-based therapy in selected hematologic malignancies, such as Hu5F9-G4 combined with rituximab in non-Hodgkin’s lymphoma (91, 148). Parallel efforts target functional reprogramming without cell elimination, as exemplified by CLEVER-1 inhibition, which aims to convert TAMs from tumor accomplices into drivers of anti-PD-1 responsiveness (151). However, clinical translation requires determining which tumors are truly TAM-dependent, which TAM program is dominant, and whether that program can be safely intercepted.

The major translational bottlenecks and biomarker-guided solutions for TAM-directed therapy are summarized in **Table 4**, and representative clinical strategies with candidate biomarkers are listed in **Supplementary Table 4**. Despite strong biological rationale, however, the path from preclinical promise to clinical benefit has been slower and less predictable than initially expected. Several TAM-directed approaches, including CCR2/CCR5 antagonists and CSF1R inhibitors, advanced into clinical testing on the basis of compelling animal data, yet their activity in patients has generally been modest or inconsistent (120, 121). The limited clinical success of some TAM-directed approaches should be interpreted as a translational barrier rather than a rejection of TAM biology as a therapeutic target. Several factors are likely involved. The myeloid compartment is redundant, and blockade of a single chemokine receptor, survival pathway, or polarization cue may be bypassed by alternative recruitment, differentiation, or survival mechanisms. TAM heterogeneity adds a second layer of complexity. Within the same lesion, macrophage subsets can differ in ontogeny, spatial niche, activation program, and therapeutic dependence; consequently, nonspecific depletion or pathway blockade may spare the subset most responsible for resistance. Safety also limits how aggressively macrophages can be targeted, because macrophages maintain immune-complex clearance, mucosal defense, tissue repair, and wound healing. This is particularly relevant for macrophage-depleting strategies and phagocytosis-checkpoint-directed agents, where systemic effects may restrict combination dosing. The most important unresolved issue is patient selection. In the absence of validated macrophage-centered biomarkers, trials may include tumors that are not primarily driven by TAM-mediated immunosuppression or that depend on different TAM states, thereby weakening efficacy signals. Modest or negative clinical results therefore argue for more precise TAM-directed therapy: pathway-matched enrollment, pharmacodynamic monitoring of myeloid remodeling, and combinations built around compensatory resistance circuits. Future development therefore needs to move beyond pan-macrophage targeting and toward strategies informed by single-cell resolution, spatial organization, and functional myeloid states.

**Table 4:** Translational bottlenecks and biomarker-guided solutions.

Translational bottleneck	Biological reason	Proposed solution	Ref.
Myeloid redundancy	Alternative recruitment or survival pathways compensate after blockade	Combine recruitment blockade with program-matched TAM-directed intervention	(120, 121)
TAM heterogeneity	Different TAM subsets have distinct dependence on CSF1R, chemokines, or stromal niches	Select patients by dominant TAM program rather than total TAM abundance	(25, 30–37)
Spatial immune exclusion	TAMs form suppressive neighborhoods with CAFs, vessels, and tumor cells	Use spatial transcriptomics and multiplex imaging	(25, 38–40)
Treatment-induced plasticity	Therapy may re-educate TAMs or enrich suppressive myeloid states	Monitor TAM dynamics before and after treatment	(153, 154)
Toxicity	Macrophages maintain homeostasis, tissue repair, and host defense	Prefer local delivery or program-selective reprogramming	(120, 148)
Lack of validated biomarkers	TAM abundance alone cannot predict dependence on TAM-mediated resistance	Develop macrophage-centered tissue and blood biomarkers	(155–157)
Inadequate trial design	Many studies enroll unselected patients	Use biomarker-guided and spatially informed combinations	(156–158)

Toward an integrated framework of TAM phenotypes, spatial niches, and reprogramming pathways. TAM-directed therapy requires a more precise definition of what is being targeted. TAMs are not a single macrophage population, but a collection of phenotypically distinct states that occupy specific niches and are maintained by different reprogramming mechanisms. The staged framework in this review highlights macrophage programs because recruitment, education, suppressive execution, and therapeutic adaptation describe how TAMs sustain ICB resistance. Yet each program is implemented by a definable macrophage phenotype, positioned within a local cellular neighborhood, and reinforced by signaling, metabolic, or epigenetic pathways. Current single-cell, spatial, and multi-omic studies support this integrated view, in which clinically actionable TAM states are defined by phenotype, location, and dominant mechanism of maintenance (13, 14, 28, 29, 159). Phenotype specifies the macrophage state, including SPP1+, TREM2+, C1QC+, interferon-responsive, or tissue-resident-like programs. Location determines whether that state is placed to exclude T cells, engage exhausted lymphocytes, remodel vasculature, or occupy stromal and perivascular niches. Mechanism defines whether the state is sustained by recruitment cues, cytokine circuits, metabolic wiring, epigenetic remodeling, or treatment-induced adaptation. In practice, therapeutic development should begin by identifying the dominant TAM state, mapping its niche and neighboring cells, defining the pathway that maintains it, and selecting combinations that target this phenotype–niche–pathway unit. This integrated framework preserves the program-based logic of the review while anchoring it in the phenotypic, spatial, and mechanistic complexity of TAM biology.

This biomarker-guided transition should not be restricted to measuring macrophage abundance. Increasingly, therapeutic resistance is being interpreted as a composite transcriptional and ecological state, shaped by tumor-intrinsic adaptation, stromal remodeling, and myeloid suppression. In prostate cancer, transcriptional programs associated with anti-androgen resistance have nominated PLK1 inhibition as a potential means of restoring enzalutamide sensitivity (103). In esophageal squamous cell carcinoma, a 23-gene model captures immune–stromal interactions that may inform therapeutic selection (160). Similarly, mesenchymal features in mismatch repair-proficient colorectal cancer have helped define subgroups with potentially actionable checkpoint dependencies (20), whereas circulating cancer-associated macrophage-like cells in NSCLC provide a functional liquid-biopsy readout that may be more informative than static enumeration of circulating tumor cells (155). Together, these observations suggest that macrophage-oriented biomarkers should be embedded within broader frameworks that integrate immune, stromal, and tumor-intrinsic mechanisms of resistance, rather than being interpreted as isolated myeloid markers. The success of TAM-directed therapies will therefore depend on the ability to navigate the marked heterogeneity of the tumor immune microenvironment through precise patient stratification (158). Broad indicators such as tumor mutation burden have been useful as initial guides for predicting responses to immune checkpoint inhibitors (156), but they do not capture the complexity of myeloid regulation. The field is consequently shifting toward higher-resolution signatures that reflect macrophage function, spatial context, and crosstalk with stromal and malignant cells. A notable example comes from hepatocellular carcinoma, where a macrophage-derived seven-gene signature was developed to predict prognosis and therapeutic response (157). This type of approach illustrates how complex macrophage-related information can be translated into clinically usable stratification tools. Importantly, monitoring myeloid function is not limited to tissue biopsies. In non-small cell lung cancer, conventional counts of circulating tumor cells often provide limited prognostic information. By contrast, cancer-associated macrophage-like cells, defined as circulating monocyte/macrophage-lineage cells that have engulfed tumor material, offer a more functional readout of host–tumor interaction. High CAML counts have been associated with substantially worse outcomes, including a reported 12-fold increase in mortality risk, suggesting that phagocytic engagement may provide a clearer indication of disease biology and therapeutic response than simple tumor-cell enumeration (155). Such biomarkers may help distinguish patients likely to benefit from ICB from those who should be directed toward alternative systemic strategies. This need is further emphasized by studies linking specific myeloid states to therapeutic failure in prostate cancer (161) and by the identification of additional resistance drivers, such as ITGB6 in head and neck malignancies (162). The biological plausibility of these approaches is supported by observations from infectious disease immunology: PD-1 inhibition has been associated with improved Mycobacterium tuberculosis clearance after restoration of IFN-γ+TNF-α+ CD4+ T-cell responses (163). This supports the broader concept that relieving inhibitory circuits may restore immune effector programs, although the cancer-specific relevance of this analogy requires direct validation.

Effective TAM-directed therapy will require spatial information, not only measurements of immune-cell abundance. High-definition spatial biology tools, including spatial transcriptomics and multiplex immunofluorescence, now enable mapping of the “neighborhoods” where TAMs interact with cancer-associated fibroblasts (CAFs) and tumor cells (116, 127). Defining these cellular interactions is clinically relevant because they may identify the immunosuppressive axes that protect residual disease. In bladder cancer, for instance, this spatial fidelity has already begun to distinguish the transcriptomic features driving response to neoadjuvant ICB, offering a blueprint for the next generation of predictive biomarkers (127, 158). Thus, spatial profiling should be used not only to describe TAM heterogeneity, but to identify the cellular neighborhoods that must be disrupted to restore immune access.

The clinical use of macrophage-based biomarkers remains limited by sampling and assay variability. TAM phenotypes are shaped by spatial heterogeneity, treatment timing, tissue processing, and the site from which a specimen is obtained; as a result, a single biopsy or single-marker readout may not identify the macrophage program that is driving resistance. The technical problem is equally important. Histology-based assays, multiplex imaging, flow cytometry, single-cell RNA sequencing, spatial transcriptomics, and circulating myeloid-cell profiling do not provide equivalent information on marker resolution, spatial organization, quantitative thresholds, or computational interpretation. Clinical translation will therefore require more than candidate markers. It will require standardized panels, reproducible scoring or analytical workflows, predefined cutoffs, and prospective validation across tumor types, therapeutic settings, and assay platforms.

Target identification alone is unlikely to be sufficient for effective TAM-directed therapy, given the redundancy and adaptability of macrophage signaling networks. In many tumors, TAM plasticity is shaped by multiple, partially overlapping pathways; therefore, blockade of a single axis may be readily compensated by alternative recruitment, survival, or polarization signals. More durable therapeutic responses may require rational co-targeting strategies that both interrupt monocyte/macrophage recruitment, for example through CCR1 inhibition, and deliver agents capable of shifting suppressive, tumor-supportive macrophage programs toward more inflammatory and tumoricidal functions (123, 137). However, the design of such combinations needs careful consideration, as standard-of-care therapies do not necessarily create an immune context that favors macrophage reactivation. Glioblastoma illustrates this challenge. Conventional radiotherapy combined with temozolomide did not increase CD163+ TAM infiltration or induce PD-1/PD-L1 upregulation. Instead, this regimen showed a tendency to enrich Foxp3+ regulatory T cells, suggesting that it may further consolidate an immunosuppressive microenvironment rather than reverse it (154). By contrast, combinations built around defined mechanisms of microenvironmental remodeling appear more promising. For example, dual VEGFA/BRAF inhibition together with PD-1 blockade has been shown to reshape the tumor milieu, in part by promoting recruitment of effector macrophage populations with antitumor activity (153). These findings underscore that TAM-directed combinations should not simply add macrophage-modulating agents to existing regimens, but should be selected according to how each component influences the broader immune ecology of the tumor.

Alongside pharmacological approaches, bioengineering strategies are expanding the therapeutic options for local macrophage modulation. Nanomedicine offers a potential solution to one of the major limitations of systemic TAM-directed intervention: off-tumor toxicity caused by interference with macrophage functions in normal tissues. Engineered extracellular vesicles and related nanoscale carriers can deliver polarity-modulating drugs directly into the tumor microenvironment, thereby enhancing local macrophage reprogramming while limiting systemic exposure. Such platforms have also been explored as a means of reversing chemoresistance by altering macrophage–tumor cell communication within the TME (147, 150). In parallel, macrophage-based cellular therapies are emerging as active therapeutic platforms rather than passive delivery systems. Inspired by the CAR-T cell paradigm, chimeric antigen receptor macrophages are being developed to combine tumor recognition, phagocytosis, and local antigen presentation. Although still at an early stage of development, CAR-Ms provide a conceptually attractive strategy for converting macrophages into effectors of tumor clearance while reinforcing local anti-tumor immunity. M1-derived extracellular vesicles may represent another biologically active approach for innate immune reprogramming within the TME (147). These vesicles could function not only as carriers, but also as sources of inflammatory signals that support a local shift toward anti-tumor macrophage states. Although these technologies remain largely preclinical, they address a central problem in the field: how to manipulate macrophage behavior at the tumor site without compromising their essential roles in host defense, tissue repair, and homeostasis elsewhere in the body. Thus, the next phase of TAM-directed therapy will likely depend not only on the discovery of new macrophage targets, but also on the development of spatially selective delivery systems, mechanism-based combinations, and engineered cellular or vesicular platforms capable of remodeling macrophage function within the tumor microenvironment.

Overall, clinical translation requires a shift from pan-macrophage targeting to program-matched, biomarker-guided, spatially informed therapy. The most informative trials will be those that prospectively define the dominant TAM resistance program, monitor macrophage state changes during treatment, and test whether functional reprogramming correlates with restored T-cell infiltration, antigen presentation, or antibody engagement. The remaining challenge is to convert TAM biology into practical criteria for patient selection, combination design, and treatment monitoring.

## 6 Conclusion

TAM-mediated ICB resistance is a staged and adaptive process, rather than a uniform suppressive phenotype. Macrophages are recruited into tumors, reshaped by local tumor and stromal signals, and then sustain resistance through intersecting immune, metabolic, stromal, phagocytic, and antibody-handling programs. These programs vary by tissue context, but they often produce recurrent suppressive effects across cancers: reduced T-cell function, impaired antigen presentation and phagocytosis, metabolic exclusion, and reinforcement of stromal immunosuppression (10, 19, 23).

The gap between preclinical rationale and clinical benefit remains substantial. Redundant myeloid pathways can compensate when a single axis is blocked (8), while macrophage plasticity may allow suppressive programs to re-emerge during treatment (9). Toxicity further limits broad disruption of macrophage biology. Equally important, the absence of validated macrophage-centered biomarkers has meant that many trials enroll patients without evidence of TAM-dependent resistance, weakening potential efficacy signals. Clinical progress will therefore require patient selection based on macrophage dependence, dominant resistance program, and spatial organization, not simply total TAM abundance.

Future work should focus on defining which macrophage states are causal drivers of resistance, which can be safely targeted, and which can be identified prospectively with spatially informed biomarkers. Only then can TAM heterogeneity be translated into practical clinical decision rules through program-matched combinations and dynamic monitoring of the myeloid compartment during treatment.

## References

[1] Zhou L Zhao T Zhang R Chen C Li J . New insights into the role of macrophages in cancer immunotherapy. Front Immunol. (2024) 15:1381225. doi: 10.3389/fimmu.2024.1381225 38605951 PMC11007015

[2] Kwantwi LB . Overcoming anti-Pd-1/Pd-L1 immune checkpoint blockade resistance: The role of macrophage, neutrophils and mast cells in the tumor microenvironment. Clin Exp Med. (2023) 23:3077–91. doi: 10.1007/s10238-023-01059-4 37022584

[3] Bi K Kashima S Camp SY Meli K Saad E Titchen BM . Myeloid cells mediate interferon-driven resistance to immunotherapy in advanced renal cell carcinoma. Immunity. (2025) 58:2814–29.e9. doi: 10.1016/j.immuni.2025.10.013 41175876 PMC12616721

[4] Yang X Deng Y Ye Y Meng J Su M Wei W . Macrophage-derived itaconate suppresses dendritic cell function to promote acquired resistance to anti-Pd-1 immunotherapy. Cancer Res. (2025) 85:1842–56. doi: 10.1158/0008-5472.can-24-2982 40036156

[5] Anandhan S Herbrich S Goswami S Guan B Chen Y Macaluso MD . Tsg-6+ cancer-associated fibroblasts modulate myeloid cell responses and impair anti-tumor response to immune checkpoint therapy in pancreatic cancer. Nat Commun. (2024) 15:5291. doi: 10.1038/s41467-024-49189-x 38987547 PMC11237123

[6] Sun K Zhang X Shi J Huang J Wang S Li X . Elevated protein lactylation promotes immunosuppressive microenvironment and therapeutic resistance in pancreatic ductal adenocarcinoma. J Clin Invest. (2025) 135:e187024. doi: 10.1172/jci187024 39883522 PMC11957693

[7] Liu T Sun T Chen X Wu J Sun X Liu X . Targeting Arpc1b overcomes immune checkpoint inhibitor resistance in glioblastoma by reversing protumorigenic macrophage polarization. Cancer Res. (2025) 85:1236–52. doi: 10.1158/0008-5472.can-24-2286 39841088

[8] Domingos-Pereira S Sathiyanadan K Polak L Haefliger JA Schmittnaegel M Ries CH . Tumor-microenvironment characterization of the Mb49 non-muscle-invasive bladder-cancer orthotopic model towards new therapeutic strategies. Int J Mol Sci. (2022) 24:123. doi: 10.3390/ijms24010123 36613562 PMC9820528

[9] Mantovani A Allavena P . The interaction of anticancer therapies with tumor-associated macrophages. J Exp Med. (2015) 212:435–45. doi: 10.1084/jem.20150295 25753580 PMC4387285

[10] Hao D Chen S . Targeting tumor-associated macrophages in non-small cell lung cancer: Mechanisms, prognosis, and therapeutic opportunities. Front Immunol. (2025) 16:1679537. doi: 10.3389/fimmu.2025.1679537 41058699 PMC12497857

[11] Zhang W Zhang W Wu H Han X . Harnessing innate immunity against glioblastoma microenvironment. Front Immunol. (2025) 16:1648601. doi: 10.3389/fimmu.2025.1648601 40787444 PMC12331708

[12] Cassetta L Pollard JW . A timeline of tumour-associated macrophage biology. Nat Rev Cancer. (2023) 23:238–57. doi: 10.1038/s41568-022-00547-1 36792751

[13] Cheng S Li Z Gao R Xing B Gao Y Yang Y . A pan-cancer single-cell transcriptional atlas of tumor infiltrating myeloid cells. Cell. (2021) 184:792–809.e23. doi: 10.1016/j.cell.2021.01.010 33545035

[14] Mulder K Patel AA Kong WT Piot C Halitzki E Dunsmore G . Cross-tissue single-cell landscape of human monocytes and macrophages in health and disease. Immunity. (2021) 54:1883–1900.e5. doi: 10.1016/j.immuni.2021.07.007 34331874

[15] Xiao M He J Yin L Chen X Zu X Shen Y . Tumor-associated macrophages: Critical players in drug resistance of breast cancer. Front Immunol. (2021) 12:799428. doi: 10.3389/fimmu.2021.799428 34992609 PMC8724912

[16] Chen L Oke T Siegel N Cojocaru G Tam AJ Blosser RL . The immunosuppressive niche of soft-tissue sarcomas is sustained by tumor-associated macrophages and characterized by intratumoral tertiary lymphoid structures. Clin Cancer Res. (2020) 26:4018–30. doi: 10.1158/1078-0432.ccr-19-3416 32332015 PMC8772618

[17] Hildebrand KM Singla AK McNeil R Marritt KL Hildebrand KN Zemp F . The Krasg12d;Trp53fl/Fl murine model of undifferentiated pleomorphic sarcoma is macrophage dense, lymphocyte poor, and resistant to immune checkpoint blockade. PLoS One. (2021) 16:e0253864. doi: 10.1371/journal.pone.0253864 34242269 PMC8270133

[18] Qian Y Galan-Cobo A Guijarro I Dang M Molkentine D Poteete A . Mct4-dependent lactate secretion suppresses antitumor immunity in Lkb1-deficient lung adenocarcinoma. Cancer Cell. (2023) 41:1363–80.e7. doi: 10.1016/j.ccell.2023.05.015 37327788 PMC11161201

[19] Novysedlak R Guney M Al Khouri M Bartolini R Koumbas Foley L Benesova I . The immune microenvironment in prostate cancer: A comprehensive review. Oncology. (2025) 103:521–45. doi: 10.1159/000541881 39380471 PMC12140600

[20] Nygaard V Ree AH Dagenborg VJ Børresen-Dale AL Edwin B Fretland ÅA . A Prrx1 signature identifies Tim-3 and Vista as potential immune checkpoint targets in a subgroup of microsatellite stable colorectal cancer liver metastases. Cancer Res Commun. (2023) 3:235–44. doi: 10.1158/2767-9764.crc-22-0295 36968142 PMC10035516

[21] Gurjao C Liu D Hofree M AlDubayan SH Wakiro I Su MJ . Intrinsic resistance to immune checkpoint blockade in a mismatch repair-deficient colorectal cancer. Cancer Immunol Res. (2019) 7:1230–36. doi: 10.1158/2326-6066.cir-18-0683 31217164 PMC6679789

[22] Li F Huang Q Luster TA Hu H Zhang H Ng WL . *In vivo* epigenetic Crispr screen identifies Asf1a as an immunotherapeutic target in Kras-mutant lung adenocarcinoma. Cancer Discov. (2020) 10:270–87. doi: 10.1158/2159-8290.cd-19-0780 31744829 PMC7007372

[23] Wang Y Liu N Guo X Han R Bai J Zhong J . The immune microenvironment in endometrial carcinoma: Mechanisms and therapeutic targeting. Front Immunol. (2025) 16:1586315. doi: 10.3389/fimmu.2025.1586315 40746533 PMC12310473

[24] Mollaoglu G Tepper A Falcomatà C Potak HT Pia L Amabile A . Ovarian cancer-derived Il-4 promotes immunotherapy resistance. Cell. (2024) 187:7492–510 e22. doi: 10.1016/j.cell.2024.10.006 39481380 PMC11682930

[25] Li Y Zheng Y Huang J Nie RC Wu QN Zuo Z . Caf-macrophage crosstalk in tumour microenvironments governs the response to immune checkpoint blockade in gastric cancer peritoneal metastases. Gut. (2025) 74:350–63. doi: 10.1136/gutjnl-2024-333617 39537239 PMC11874311

[26] Stewart BJ Fergie M Young MD Jones C Sachdeva A Blain A . Spatial and molecular profiling of the mononuclear phagocyte network in classic Hodgkin lymphoma. Blood. (2023) 141:2343–58. doi: 10.1182/blood.2022015575 36758207

[27] Chao NN Zhang L . Elucidating cellular origins and Tme dynamic evolution in Nsclc through multi-omics technologies. Biochim Biophys Acta (BBA) - Rev Cancer. (2025) 1880:189425. doi: 10.1016/j.bbcan.2025.189425 40840819

[28] Ma RY Black A Qian BZ . Macrophage diversity in cancer revisited in the era of single-cell omics. Trends Immunol. (2022) 43:546–63. doi: 10.1016/j.it.2022.04.008 35690521

[29] Coulton A Murai J Qian D Thakkar K Lewis CE Litchfield K . Using a pan-cancer atlas to investigate tumour associated macrophages as regulators of immunotherapy response. Nat Commun. (2024) 15:5665. doi: 10.1038/s41467-024-49885-8 38969631 PMC11226649

[30] Ma Q Wang J Jiang Q . Ca9+ cancer-associated fibroblasts cooperate with Spp1+ tumor-associated macrophages driving immune resistance in triple-negative breast cancer. Cell Mol Life Sci: CMLS. (2026) 83:54. doi: 10.1007/s00018-025-06056-2 41511527 PMC12819957

[31] Zhao R Pan Z Qiu J Li B Qi Y Gao Z . Blocking Itga5 potentiates the efficacy of anti-Pd-1 therapy on glioblastoma by remodeling tumor-associated macrophages. Cancer Commun (London England). (2025) 45:677–701. doi: 10.1002/cac2.70016 40084746 PMC12187582

[32] Jiang W Liu L Xu Z Qiu Y Zhang B Cheng J . Spp1+ tumor-associated macrophages drive immunotherapy resistance via Cd8+ T-cell dysfunction in clear-cell renal cell carcinoma. Cancer Immunol Res. (2025) 13:1533–46. doi: 10.1158/2326-6066.cir-24-1146 40680256 PMC12485372

[33] Zhang S Peng W Wang H Xiang X Ye L Wei X . C1q+ tumor-associated macrophages contribute to immunosuppression through fatty acid metabolic reprogramming in Malignant pleural effusion. J Immunother Cancer. (2023) 11:e007441. doi: 10.1136/jitc-2023-007441 37604643 PMC10445384

[34] Binnewies M Pollack JL Rudolph J Dash S Abushawish M Lee T . Targeting Trem2 on tumor-associated macrophages enhances immunotherapy. Cell Rep. (2021) 37:109844. doi: 10.1016/j.celrep.2021.109844 34686340

[35] Wu Q Zhou W Yin S Zhou Y Chen T Qian J . Blocking triggering receptor expressed on myeloid cells-1-positive tumor-associated macrophages induced by hypoxia reverses immunosuppression and anti-programmed cell death ligand 1 resistance in liver cancer. Hepatol (Baltimore Md). (2019) 70:198–214. doi: 10.1002/hep.30593 30810243 PMC6618281

[36] Li X Poire A Jeong KJ Zhang D Ozmen TY Chen G . C5ar1 inhibition reprograms tumor associated macrophages and reverses Parp inhibitor resistance in breast cancer. Nat Commun. (2024) 15:4485. doi: 10.1038/s41467-024-48637-y 38802355 PMC11130309

[37] You L Wang Q Zhang T Xiao H Lv M Lv H . Usp14-Imp2-Cxcl2 axis in tumor-associated macrophages facilitates resistance to anti-Pd-1 therapy in gastric cancer by recruiting myeloid-derived suppressor cells. Oncogene. (2025) 44:2413–26. doi: 10.1038/s41388-025-03425-w 40269263

[38] Wang Z Xu W Zhao L Zhong L Yu W Wang S . Il4i1^+^ macrophages and Tdo2^+^ myofibroblasts drive Ahr-mediated immunosuppression and ferroptosis resistance in solid predominant lung adenocarcinoma. Adv Sci (Weinheim Baden-Wurttemberg Germany). (2026) 13:e13606. doi: 10.1002/advs.202513606 41431173 PMC12955909

[39] Al-Janabi H Moyes K Allen R Fisher M Crespo M Gurel B . Targeting a Sting agonist to perivascular macrophages in prostate tumors delays resistance to androgen deprivation therapy. J Immunother Cancer. (2024) 12:e009368. doi: 10.1136/jitc-2024-009368 39060021 PMC11284826

[40] Ning J Ye Y Shen H Zhang R Li H Song T . Macrophage-coated tumor cluster aggravates hepatoma invasion and immunotherapy resistance via generating local immune deprivation. Cell Rep Med. (2024) 5:101505. doi: 10.1016/j.xcrm.2024.101505 38614095 PMC11148514

[41] Xu XW Zhang Y Zhao CW Wu H Zhang MT Ma YT . Lenvatinib-resistant liver cancer-derived Hsp90α-containing extracellular vesicles enhance drug resistance via macrophage Cxcl8 secretion. Immunol Lett. (2026) 278:107122. doi: 10.1016/j.imlet.2025.107122 41349809

[42] Chryplewicz A Scotton J Tichet M Zomer A Shchors K Joyce JA . Cancer cell autophagy, reprogrammed macrophages, and remodeled vasculature in glioblastoma triggers tumor immunity. Cancer Cell. (2022) 40:1111–27 e9. doi: 10.1016/j.ccell.2022.08.014 36113478 PMC9580613

[43] Roehle K Qiang L Ventre KS Heid D Ali LR Lenehan P . Ciap1/2 antagonism eliminates Mhc class I-negative tumors through T cell-dependent reprogramming of mononuclear phagocytes. Sci Transl Med. (2021) 13:eabf5058. doi: 10.1126/scitranslmed.abf5058 34011631 PMC8406785

[44] Zhang JF Liu T . Tyro3 and Cdk9 as biomarkers for drug resistance to breast cancer anti-Pd-1 therapies. Zhonghua Zhong Liu Za Zhi [Chinese J Oncol]. (2023) 45:651–56. doi: 10.3760/cma.j.cn112152-20210223-00161 37580269

[45] Jiang W Guan B Sun H Mi Y Cai S Wan R . Wnt11 promotes immune evasion and resistance to anti-Pd-1 therapy in liver metastasis. Nat Commun. (2025) 16:1429. doi: 10.1038/s41467-025-56714-z 39920102 PMC11806061

[46] Aslan K Turco V Blobner J Sonner JK Liuzzi AR Núñez NG . Heterogeneity of response to immune checkpoint blockade in hypermutated experimental gliomas. Nat Commun. (2020) 11:931. doi: 10.1038/s41467-020-14642-0 32071302 PMC7028933

[47] Sun L Chu X Kong T Chen X Ru J Gao Q . An SPP1-SOCS1 pathway constrains interferon responses in tumor-associated macrophages and shapes an immunosuppressive tumor microenvironment. Immunity. (2026) 59:1422–37.e9. doi: 10.1016/j.immuni.2026.04.001 42049035

[48] Xu H Xue S Sun Y Ma J Li S Wang Y . Creb3l1 facilitates pancreatic tumor progression and reprograms intratumoral tumor-associated macrophages to shape an immunotherapy-resistance microenvironment. J Immunother Cancer. (2025) 13:e010029. doi: 10.1136/jitc-2024-010029 39762079 PMC11749327

[49] Ni J Li X Shi Z Guo B Duan Y An Y . Peak1 promotes prostate cancer progression and docetaxel resistance by mediating the polarization of tumor-associated macrophages. Eur J Med Res. (2026) 31:41. doi: 10.1186/s40001-025-03568-2 41495812 PMC12781285

[50] Lin H Fu L Zhou X Yu A Chen Y Liao W . Lrp1 induces anti-Pd-1 resistance by modulating the Dll4-Notch2-Ccl2 axis and redirecting M2-like macrophage polarisation in bladder cancer. Cancer Lett. (2024) 593:216807. doi: 10.1016/j.canlet.2024.216807 38462037

[51] Chen H Yang W Xue X Li Y Jin Z Ji Z . Integrated analysis revealed an inflammatory cancer-associated fibroblast-based subtypes with promising implications in predicting the prognosis and immunotherapeutic response of bladder cancer patients. Int J Mol Sci. (2022) 23:15970. doi: 10.3390/ijms232415970 36555612 PMC9781727

[52] Zhang J Dong Y Yu S Hu K Zhang L Xiong M . Il-4/Il-4r axis signaling drives resistance to immunotherapy by inducing the upregulation of Fcγ receptor Iib in M2 macrophages. Cell Death Dis. (2024) 15:500. doi: 10.1038/s41419-024-06875-4 39003253 PMC11246528

[53] Zhang Y Gao Y Wang J Tan X Liang Y Yu W . Connexin 43 deficiency confers resistance to immunotherapy in lung cancer via inhibition of the cyclic GMP-AMP synthase-stimulator of interferon genes pathway. J Cell Mol Med. (2024) 28:e70211. doi: 10.1111/jcmm.70211 39592436 PMC11598135

[54] Tao Y Tian C Qi S Jia Z Xu Z Meng J . Targeting both death and paracaspase domains of Malt1 with antisense oligonucleotides overcomes resistance to immune-checkpoint inhibitors. Nat Cancer. (2025) 6:702–17. doi: 10.1038/s43018-025-00930-5 40075237

[55] Troiani T Giunta EF Tufano M Vigorito V Arrigo P Argenziano G . Alternative macrophage polarisation associated with resistance to anti-Pd1 blockade is possibly supported by the splicing of Fkbp51 immunophilin in melanoma patients. Br J Cancer. (2020) 122:1782–90. doi: 10.1038/s41416-020-0840-8 32317723 PMC7283486

[56] Chen Y Jia H Zhang X Zhao H Xiao Y Li N . Disruption of Gpsm1/Csf1 signaling reprograms tumor-associated macrophages to overcome anti-Pd-1 resistance in colorectal cancer. J Immunother Cancer. (2025) 13:e010826. doi: 10.1136/jitc-2024-010826 40010765 PMC12083360

[57] Zhang W Li JB Liu HM Wang KM Xiao BL Wang YM . Perk+ macrophages drive immunotherapy resistance in lymph node metastases of oral squamous cell carcinoma. Clin Cancer Res. (2025) 31:1894–911. doi: 10.1158/1078-0432.ccr-24-3135 40036693

[58] Grant M Cipriani B Corbin A Miller D Naylor A Hughes S . Low pH, high stakes: A narrative review exploring the acid-sensing GPR65 pathway as a novel approach in renal cell carcinoma. Cancers (Basel). (2025) 17:3883. doi: 10.3390/cancers17233883 41375084 PMC12691181

[59] Huang M . Histone lactylation in gastrointestinal cancers: Developing immunotherapeutic drugs targeting epigenetics. Arch Pharmacal Res. (2025) 48:831–42. doi: 10.1007/s12272-025-01570-0 40976819

[60] Xin L Liu J Lai JY Xu HS Fan LJ Zou YH . Methionine restriction promotes the polarization of macrophages towards M1 and the immunotherapy effect of Pd-L1/Pd-1 blockades by inhibiting the secretion of Mif by gastric carcinoma cells. Transl Oncol. (2025) 51:102181. doi: 10.1016/j.tranon.2024.102181 39541710 PMC11600783

[61] Jiang Z Lim SO Yan M Hsu JL Yao J Wei Y . Tyro3 induces anti-Pd-1/Pd-L1 therapy resistance by limiting innate immunity and tumoral ferroptosis. J Clin Invest. (2021) 131:e139434. doi: 10.1172/jci139434 33855973 PMC8262501

[62] Quaranta V Rainer C Nielsen SR Raymant ML Ahmed MS Engle DD . Macrophage-derived granulin drives resistance to immune checkpoint inhibition in metastatic pancreatic cancer. Cancer Res. (2018) 78:4253–69. doi: 10.1158/0008-5472.can-17-3876 29789416 PMC6076440

[63] Chen Y Liu X Chen J Kuang L Zhang N Li D . Macrophage Ccl7 promotes resistance to immunotherapy for colorectal cancer by regulating the infiltration of macrophages and CD8+ T cells. J Immunother Cancer. (2025) 13:e013027. doi: 10.1136/jitc-2025-013027 41290259 PMC12645621

[64] Han N Baghdadi M Ishikawa K Endo H Kobayashi T Wada H . Enhanced Il-34 expression in nivolumab-resistant metastatic melanoma. Inflammation Regener. (2018) 38:3. doi: 10.1186/s41232-018-0060-2 29515691 PMC5836392

[65] Baghdadi M Wada H Nakanishi S Abe H Han N Putra WE . Chemotherapy-induced Il34 enhances immunosuppression by tumor-associated macrophages and mediates survival of chemoresistant lung cancer cells. Cancer Res. (2016) 76:6030–42. doi: 10.1158/0008-5472.can-16-1170 27550451

[66] Igarashi Y Seino KI . Role of Il-34 in tumors and its application to regulate inflammation. Cancer Sci. (2025) 116:1164–70. doi: 10.1111/cas.70020 39951014 PMC12044644

[67] Wang JC Chen DP Lu SX Chen JB Wei Y Liu XC . Pim2 expression induced by proinflammatory macrophages suppresses immunotherapy efficacy in hepatocellular carcinoma. Cancer Res. (2022) 82:3307–20. doi: 10.1158/0008-5472.can-21-3899 35802648 PMC9478531

[68] Ning J Hou X Hao J Zhang W Shi Y Huang Y . Mettl3 inhibition induced by M2 macrophage-derived extracellular vesicles drives anti-Pd-1 therapy resistance via M6a-Cd70-mediated immune suppression in thyroid cancer. Cell Death Differ. (2023) 30:2265–79. doi: 10.1038/s41418-023-01217-x 37648786 PMC10589295

[69] Morrissey SM Yan J . Exosomal Pd-L1: Roles in tumor progression and immunotherapy. Trends Cancer. (2020) 6:550–8. doi: 10.1016/j.trecan.2020.03.002 32610067 PMC7330176

[70] Lamorte S Quevedo R Jin R Neufeld L Liu ZQ Ciudad MT . Lymph node macrophages drive immune tolerance and resistance to cancer therapy by induction of the immune-regulatory cytokine Il-33. Cancer Cell. (2025) 43:955–69:e10. doi: 10.1016/j.ccell.2025.02.017 40054466 PMC12074877

[71] Pu Y Ji Q . Tumor-associated macrophages regulate Pd-1/Pd-L1 immunosuppression. Front Immunol. (2022) 13:874589. doi: 10.3389/fimmu.2022.874589 35592338 PMC9110638

[72] Shinchi Y Komohara Y Yonemitsu K Sato K Ohnishi K Saito Y . Accurate expression of Pd-L1/L2 in lung adenocarcinoma cells: A retrospective study by double immunohistochemistry. Cancer Sci. (2019) 110:2711–21. doi: 10.1111/cas.14128 31294893 PMC6726681

[73] Yu Y Liang Y Xie F Zhang Z Zhang P Zhao X . Tumor-associated macrophage enhances Pd-L1-mediated immune escape of bladder cancer through Pkm2 dimer-Stat3 complex nuclear translocation. Cancer Lett. (2024) 593:216964. doi: 10.1016/j.canlet.2024.216964 38762193

[74] Kasikara C Kumar S Kimani S Tsou WI Geng K Davra V . Phosphatidylserine sensing by Tam receptors regulates Akt-dependent chemoresistance and Pd-L1 expression. Mol Cancer Res: MCR. (2017) 15:753–64. doi: 10.1158/1541-7786.mcr-16-0350 28184013 PMC8363069

[75] Dong H Han J Chen X Sun H Han M Wang W . Lncrna Znf649-As1 promotes trastuzumab resistance and Tam-dependent Pd-L1 expression in breast cancer by regulating Exoc7 alternative splicing. Arch Biochem Biophys. (2024) 761:110128. doi: 10.1016/j.abb.2024.110128 39159899

[76] Sugiura D Maruhashi T Okazaki IM Shimizu K Maeda TK Takemoto T . Restriction of PD-1 function by cis-PD-L1/CD80 interactions is required for optimal T cell responses. Science. (2019) 364:558–66. doi: 10.1126/science.aav7062 31000591

[77] Zippelius A Schreiner J Herzig P Müller P . Induced Pd-L1 expression mediates acquired resistance to agonistic anti-Cd40 treatment. Cancer Immunol Res. (2015) 3:236–44. doi: 10.1158/2326-6066.cir-14-0226 25623164

[78] Zhang T Yu H Dai X Zhang X . Cmtm6 and Cmtm4 as two novel regulators of Pd-L1 modulate the tumor microenvironment. Front Immunol. (2022) 13:971428. doi: 10.3389/fimmu.2022.971428 35958549 PMC9359082

[79] Wu X Lan X Hu W Zhang W Lai X Xu S . Cmtm6 expression in M2 macrophages is a potential predictor of Pd-1/Pd-L1 inhibitor response in colorectal cancer. Cancer Immunol Immunother: CII. (2021) 70:3235–48. doi: 10.1007/s00262-021-02931-6 33818637 PMC8505364

[80] Sun J Lu Q Sanmamed MF Wang J . Siglec-15 as an emerging target for next-generation cancer immunotherapy. Clin Cancer Res. (2021) 27:680–8. doi: 10.1158/1078-0432.ccr-19-2925 32958700 PMC9942711

[81] Hayashi F Akagi K Taniguchi H Matsutake T Kawahara H Sekine I . Tim-3 expression induces resistance to Pd-1 inhibitor in G-Csf-producing lung spindle cell carcinoma: A case report. Thorac Cancer. (2023) 14:3556–60. doi: 10.1111/1759-7714.15149 37926435 PMC10733156

[82] Karihtala K Leivonen SK Brück O Karjalainen-Lindsberg ML Mustjoki S Pellinen T . Prognostic impact of tumor-associated macrophages on survival is checkpoint dependent in classical Hodgkin lymphoma. Cancers (Basel). (2020) 12:877. doi: 10.3390/cancers12040877 32260340 PMC7225916

[83] Miyauchi S Sanders PD Guram K Kim SS Paolini F Venuti A . Hpv16 E5 mediates resistance to Pd-L1 blockade and can be targeted with rimantadine in head and neck cancer. Cancer Res. (2020) 80:732–46. doi: 10.1158/0008-5472.can-19-1771 31848196 PMC7024675

[84] Paczkowska J Tang M Wright KT Song L Luu K Shanmugam V . Cancer-specific innate and adaptive immune rewiring drives resistance to Pd-1 blockade in classic Hodgkin lymphoma. Nat Commun. (2024) 15:10740. doi: 10.1038/s41467-024-54512-7 39737927 PMC11686379

[85] Zhang X Lao M Sun K Yang H He L Liu X . Sphingolipid synthesis in tumor-associated macrophages confers immunotherapy resistance in hepatocellular carcinoma. Sci Adv. (2025) 11:eadv0558. doi: 10.1126/sciadv.adv0558 40397754 PMC12094245

[86] Campesato LF Budhu S Tchaicha J Weng CH Gigoux M Cohen IJ . Blockade of the Ahr restricts a Treg-macrophage suppressive axis induced by L-kynurenine. Nat Commun. (2020) 11:4011. doi: 10.1038/s41467-020-17750-z 32782249 PMC7419300

[87] Ding W Mo J Su Y Zhang Q Sun D Yao X . Metabolic reprogramming of tumor-associated macrophages via adenosine-a(2a)R signaling drives cross-resistance in non-small cell lung cancer. Drug Resist Updates. (2025) 82:101272. doi: 10.1016/j.drup.2025.101272 40618433

[88] Scolaro T Manco M Pecqueux M Amorim R Trotta R Van Acker HH . Nucleotide metabolism in cancer cells fuels a udp-driven macrophage cross-talk, promoting immunosuppression and immunotherapy resistance. Nat Cancer. (2024) 5:1206–26. doi: 10.1038/s43018-024-00771-8 38844817 PMC11358017

[89] Li Y Wang L . Bidirectional regulation in the tumour microenvironment: The interaction between tumour-associated macrophages and T cells reshapes the paradigm of cancer immunotherapy. Immunology. (2026) 178:1–22. doi: 10.1111/imm.70103 41498784

[90] Yang M Wang B Hou W Zeng H He W Zhang XK . NAD+ metabolism enzyme nnmt in cancer-associated fibroblasts drives tumor progression and resistance to immunotherapy by modulating macrophages in urothelial bladder cancer. J Immunother Cancer. (2024) 12:e009281. doi: 10.1136/jitc-2024-009281 39067875 PMC11284830

[91] Advani R Flinn I Popplewell L Forero A Bartlett NL Ghosh N . Cd47 blockade by hu5f9-g4 and rituximab in non-hodgkin's lymphoma. N Engl J Med. (2018) 379:1711–21. doi: 10.1056/NEJMoa1807315 30380386 PMC8058634

[92] Meng Z Gao X Tang X Fan J Sheu WC Tu Z . Signal transducer nanoparticles enable siglec-10/g blockade immunotherapy for breast cancer treatment. Adv Mater (Deerfield Beach Fla). (2025) 37:e2502758. doi: 10.1002/adma.202502758 40619860

[93] Hirade K Tanaka N Kajino T Adachi Y Kimura R Kasuya H . Inhibiting kras with cd47 and immune checkpoint overcomes intrinsic resistance to combined kras and immune checkpoint inhibitor therapy. Cell Rep Med. (2025) 6:102317. doi: 10.1016/j.xcrm.2025.102317 40885190 PMC12490217

[94] Lin H Kryczek I Li S Green MD Ali A Hamasha R . Stanniocalcin 1 is a phagocytosis checkpoint driving tumor immune resistance. Cancer Cell. (2021) 39:480–93:e6. doi: 10.1016/j.ccell.2020.12.023 33513345 PMC8044011

[95] Wu SY Zhu YY Sun JL Wang CY Wang YL Nie YY . Targeting tumor-intrinsic bcl9 reverses immunotherapy resistance by eliciting macrophage-mediated phagocytosis and antigen presentation. Nat Commun. (2025) 16:10039. doi: 10.1038/s41467-025-65945-z 41249197 PMC12623502

[96] Arlauckas SP Garris CS Kohler RH Kitaoka M Cuccarese MF Yang KS . *In vivo* imaging reveals a tumor-associated macrophage-mediated resistance pathway in anti-pd-1 therapy. Sci Transl Med. (2017) 9:eaal3604. doi: 10.1126/scitranslmed.aal3604 28490665 PMC5734617

[97] Wang C Chen Q Chen M Guo S Hou P Zou Y . Interaction of glioma-associated microglia/macrophages and anti-Pd1 immunotherapy. Cancer Immunol Immunother: CII. (2023) 72:1685–98. doi: 10.1007/s00262-022-03358-3 36624155 PMC10992854

[98] Xu L Shen M Chen X Yang DR Tsai Y Keng PC . *In vitro*-induced M2 type macrophages induces the resistance of prostate cancer cells to cytotoxic action of Nk cells. Exp Cell Res. (2018) 364:113–23. doi: 10.1016/j.yexcr.2018.01.041 29408565

[99] Minowa T Hirohashi Y Murata K Sasaki K Handa T Nakatsugawa M . Fusion with type 2 macrophages induces melanoma cell heterogeneity that potentiates immunological escape from cytotoxic T lymphocytes. J Pathol. (2023) 260:304–16. doi: 10.1002/path.6083 37138382

[100] Jin R Neufeld L McGaha TL . Linking macrophage metabolism to function in the tumor microenvironment. Nat Cancer. (2025) 6:239–52. doi: 10.1038/s43018-025-00909-2 39962208

[101] Zhang D Tang Z Huang H Zhou G Cui C Weng Y . Metabolic regulation of gene expression by histone lactylation. Nature. (2019) 574:575–80. doi: 10.1038/s41586-019-1678-1 31645732 PMC6818755

[102] De Leo A Ugolini A Yu X Scirocchi F Scocozza D Peixoto B . Glucose-driven histone lactylation promotes the immunosuppressive activity of monocyte-derived macrophages in glioblastoma. Immunity. (2024) 57:1105–1123.e8. doi: 10.1016/j.immuni.2024.04.006 38703775 PMC11114377

[103] Zhuang R Xie R Peng S Zhou Q Lin W Ou Y . An anti-androgen resistance-related gene signature acts as a prognostic marker and increases enzalutamide efficacy via Plk1 inhibition in prostate cancer. J Transl Med. (2025) 23:480. doi: 10.1186/s12967-025-06457-8 40289088 PMC12034143

[104] Xu P Yang JC Chen B Nip C Van Dyke JE Zhang X . Androgen receptor blockade resistance with enzalutamide in prostate cancer results in immunosuppressive alterations in the tumor immune microenvironment. J Immunother Cancer. (2023) 11:e006581. doi: 10.1136/jitc-2022-006581 37147019 PMC10163595

[105] Lyu X Wang P Qiao Q Jiang Y . Genomic stratification based on microenvironment immune types and Pd-L1 for tailoring therapeutic strategies in bladder cancer. BMC Cancer. (2021) 21:646. doi: 10.1186/s12885-021-08350-1 34059019 PMC8166145

[106] Wu C Qiu Y Zhang R Li X Liang H Wang M . Association of peripheral basophils with tumor M2 macrophage infiltration and outcomes of the anti-Pd-1 inhibitor plus chemotherapy combination in advanced gastric cancer. J Transl Med. (2022) 20:386. doi: 10.1186/s12967-022-03598-y 36058929 PMC9441040

[107] Huang Z Li L Zhao X Jin H Shen M Li B . Scrna-seq reveals that Vegf signaling mediates the response to neoadjuvant anlotinib combined with Pd-1 blockade therapy in non-small cell lung cancer. J Transl Med. (2025) 23:478. doi: 10.1186/s12967-025-06485-4 40281576 PMC12032801

[108] Kim SR Chun SH Kim JR Kim SY Seo JY Jung CK . The implications of clinical risk factors, Car index, and compositional changes of immune cells on hyperprogressive disease in non-small cell lung cancer patients receiving immunotherapy. BMC Cancer. (2021) 21:19. doi: 10.1186/s12885-020-07727-y 33402127 PMC7786505

[109] Zhu HZ Zhou WJ Wan YF Ge K Lu J Jia CK . Downregulation of orosomucoid 2 acts as a prognostic factor associated with cancer-promoting pathways in liver cancer. World J Gastroenterol. (2020) 26:804–17. doi: 10.3748/wjg.v26.i8.804 32148378 PMC7052533

[110] Tufail M Jiang CH Li N . Immune evasion in cancer: Mechanisms and cutting-edge therapeutic approaches. Signal Transduction Targeted Ther. (2025) 10:227. doi: 10.1038/s41392-025-02280-1 40739089 PMC12311175

[111] Endo E Okayama H Saito K Nakajima S Yamada L Ujiie D . A tgfβ-dependent stromal subset underlies immune checkpoint inhibitor efficacy in DNA mismatch repair-deficient/microsatellite instability-high colorectal cancer. Mol Cancer Res MCR. (2020) 18:1402–13. doi: 10.1158/1541-7786.mcr-20-0308 32493700

[112] Petroni G Galassi C Gouin KH Chen HH Buqué A Bloy N . Il-17a-secreting γδ T cells promote resistance to cdk4/cdk6 inhibitors in hr(+)her2(-) breast cancer via cx3cr1(+) macrophages. Nat Cancer. (2025) 6:1656–75. doi: 10.1038/s43018-025-01007-z 40624238

[113] Zheng G Guo Z Li W Xi W Zuo B Zhang R . Interaction between hla-g and nk cell receptor kir2dl4 orchestrates her2-positive breast cancer resistance to trastuzumab. Signal Transduction Targeted Ther. (2021) 6:236. doi: 10.1038/s41392-021-00629-w 34158475 PMC8219715

[114] Qiu P Guo Q Yao Q Chen J Lin J . Characterization of exosome-related gene risk model to evaluate the tumor immune microenvironment and predict prognosis in triple-negative breast cancer. Front Immunol. (2021) 12:736030. doi: 10.3389/fimmu.2021.736030 34659224 PMC8517454

[115] Zhang W Zhang Q Yang N Shi Q Su H Lin T . Crosstalk between il-15rα(+) tumor-associated macrophages and breast cancer cells reduces cd8(+) T cell recruitment. Cancer Commun (London England). (2022) 42:536–57. doi: 10.1002/cac2.12311 35615815 PMC9198341

[116] Ge Q Meng J Wang Z Anwaier A Lu J Tian X . Spatially segregated apoe(+) macrophages restrict immunotherapy efficacy in clear cell renal cell carcinoma. Theranostics. (2025) 15:5312–36. doi: 10.7150/thno.109097 40303328 PMC12036886

[117] Gordon SR Maute RL Dulken BW Hutter G George BM McCracken MN . PD-1 expression by tumour-associated macrophages inhibits phagocytosis and tumour immunity. Nature. (2017) 545:495–9. doi: 10.1038/nature22396 28514441 PMC5931375

[118] Kzhyshkowska J Shen J Larionova I . Targeting of tams: Can we be more clever than cancer cells? Cell Mol Immunol. (2024) 21:1376–409. doi: 10.1038/s41423-024-01232-z 39516356 PMC11607358

[119] Reiss KA Angelos MG Dees EC Yuan Y Ueno NT Pohlmann PR . CAR-macrophage therapy for HER2-overexpressing advanced solid tumors: A phase 1 trial. Nat Med. (2025) 31:1171–82. doi: 10.1038/s41591-025-03495-z 39920391

[120] Cao J Chow L Dow S . Strategies to overcome myeloid cell induced immune suppression in the tumor microenvironment. Front Oncol. (2023) 13:1116016. doi: 10.3389/fonc.2023.1116016 37114134 PMC10126309

[121] Liu F Li X Zhang Y Ge S Shi Z Liu Q . Targeting tumor-associated macrophages to overcome immune checkpoint inhibitor resistance in hepatocellular carcinoma. J Exp Clin Cancer Res CR. (2025) 44:227. doi: 10.1186/s13046-025-03490-9 40764998 PMC12323087

[122] Fan TW Higashi RM Song H Daneshmandi S Mahan AL Purdom MS . Innate immune activation by checkpoint inhibition in human patient-derived lung cancer tissues. eLife. (2021) 10:e69578. doi: 10.7554/eLife.69578 34406120 PMC8476122

[123] Su X Huang R Kang D Wang J Li L Zou Z . Blocking ccr1(+) macrophages overcomes resistance to immune checkpoint inhibitors in melanoma. Cell Commun Signaling CCS. (2025) 23:475. doi: 10.1186/s12964-025-02444-0 41184864 PMC12581358

[124] Park N Jeon SH Kim Y Kim S Kim IA . Akt inhibition enhances the antitumor efficacy of immune checkpoint blockades and radiotherapy in a syngeneic breast cancer model. Mol Ther Oncol. (2025) 33:201087. doi: 10.1016/j.omton.2025.201087 41378083 PMC12686700

[125] Miyazaki T Ishikawa E Matsuda M Sugii N Kohzuki H Akutsu H . Infiltration of cd163-positive macrophages in glioma tissues after treatment with anti-pd-l1 antibody and role of pi3kγ inhibitor as a combination therapy with anti-pd-l1 antibody in *in vivo* model using temozolomide-resistant murine glioma-initiating cells. Brain Tumor Pathol. (2020) 37:41–9. doi: 10.1007/s10014-020-00357-z 31980975

[126] Han MG Jang BS Kang MH Na D Kim IA . Pi3kγδ inhibitor plus radiation enhances the antitumour immune effect of pd-1 blockade in syngenic murine breast cancer and humanised patient-derived xenograft model. Eur J Cancer (Oxford Engl 1990). (2021) 157:450–63. doi: 10.1016/j.ejca.2021.08.029 34601286

[127] Chen H Jin Z Peng Y Li Y Li Z Zhang X . Malevolent alliance of MYBL2hi cancer stem cell and SPP1+ macrophage confers resistance to neoadjuvant immunotherapy in bladder cancer. J Immunother Cancer. (2025) 13:e011319. doi: 10.1136/jitc-2024-011319 40639849 PMC12258365

[128] Trotta R Rivis S Zhao S Orban MP Trusso Cafarello S Charatsidou I . Activated T cells break tumor immunosuppression by macrophage reeducation. Cancer Discov. (2025) 15:1410–36. doi: 10.1158/2159-8290.cd-24-0415 40094380 PMC12223510

[129] Tong L Li J Li Q Wang X Medikonda R Zhao T . Act001 reduces the expression of pd-l1 by inhibiting the phosphorylation of stat3 in glioblastoma. Theranostics. (2020) 10:5943–56. doi: 10.7150/thno.41498 32483429 PMC7254983

[130] Chen Q Xu Y Wang Y Zheng S Yao T Qiu J . Bacteria and tumor debris induced pancreatic cancer progression via the nf-κb signaling pathway. Cancer Lett. (2025) 632:217964. doi: 10.1016/j.canlet.2025.217964 40752749

[131] Shi S Ou X Liu C Li R Zheng Q Hu L . Nf-κb signaling and the tumor microenvironment in osteosarcoma: Implications for immune evasion and therapeutic resistance. Front Immunol. (2025) 16:1518664. doi: 10.3389/fimmu.2025.1518664 39949765 PMC11821961

[132] Leshem R Sefton KN Wong CW Lin IH Isaac DT Niepel M . Combined parp14 inhibition and pd-1 blockade promotes cytotoxic T cell quiescence and modulates macrophage polarization in relapsed melanoma. J Immunother Cancer. (2025) 13:e010683. doi: 10.1136/jitc-2024-010683 39870492 PMC11772928

[133] Gao W Long X Lin X Deng K Li D Huang M . Targeting mesenchymal monocyte-derived macrophages to enhance the sensitivity of glioblastoma to temozolomide by inhibiting tnf/celsr2/p65/kla-hdac1/epas1 axis. J Adv Res. (2026) 80:925–41. doi: 10.1016/j.jare.2025.05.032 40373963 PMC12869204

[134] Dang W Xiao J Ma Q Miao J Cao M Chen L . Combination of p38 mapk inhibitor with pd-l1 antibody effectively prolongs survivals of temozolomide-resistant glioma-bearing mice via reduction of infiltrating glioma-associated macrophages and pd-l1 expression on resident glioma-associated microglia. Brain Tumor Pathol. (2021) 38:189–200. doi: 10.1007/s10014-021-00404-3 34231121

[135] Murphy S Rahmy S Gan D Liu G Zhu Y Manyak M . Ketogenic diet alters the epigenetic and immune landscape of prostate cancer to overcome resistance to immune checkpoint blockade therapy. Cancer Res. (2024) 84:1597–612. doi: 10.1158/0008-5472.can-23-2742 38588411 PMC11096030

[136] Peng S Wu M Yan Q Xu G Xie Y Tang G . Disrupting edem3-induced m2-like macrophage trafficking by glucose restriction overcomes resistance to pd-1/pd-l1 blockade. Clin Transl Med. (2025) 15:e70161. doi: 10.1002/ctm2.70161 39754316 PMC11702414

[137] Hao X Zheng Z Liu H Zhang Y Kang J Kong X . Inhibition of apoc1 promotes the transformation of m2 into m1 macrophages via the ferroptosis pathway and enhances anti-pd1 immunotherapy in hepatocellular carcinoma based on single-cell rna sequencing. Redox Biol. (2022) 56:102463. doi: 10.1016/j.redox.2022.102463 36108528 PMC9482117

[138] Wang YZ Gao N Lin Z Wang SH Ai S Wei Z . Dissection of immunotherapeutic predictive versus prognostic transcriptional programs identifies slc22a5-centric carnitine metabolism-driven resistance to anti-pd-(l)1 treatment in non-small cell lung cancer. Drug Resist Updates. (2026) 84:101313. doi: 10.1016/j.drup.2025.101313 41045681

[139] Pi YN Qi WC Xia BR Lou G Jin WL . Long non-coding rnas in the tumor immune microenvironment: Biological properties and therapeutic potential. Front Immunol. (2021) 12:697083. doi: 10.3389/fimmu.2021.697083 34295338 PMC8290853

[140] Zhao R Li B Zhang S He Z Pan Z Guo Q . The n(6)-methyladenosine-modified pseudogene hspa7 correlates with the tumor microenvironment and predicts the response to immune checkpoint therapy in glioblastoma. Front Immunol. (2021) 12:653711. doi: 10.3389/fimmu.2021.653711 34354698 PMC8329659

[141] Skossyrskiy VS Kurdina NA Kuzovkova VS Boot MS Zelenchenkova PI Popova EO . Cyclooxygenase-2 as a potential therapeutic target in the treatment of chemoresistant glioblastomas. Med Oncol (Northwood London England). (2025) 42:530. doi: 10.1007/s12032-025-03000-z 41137875

[142] Cheng Y Han X Lai X Wei X . Liposomal honokiol inhibits non-small cell lung cancer progression and enhances pd-1 blockade via suppressing m2 macrophages polarization. Phytomed Int J Phytother Phytopharmacology. (2024) 135:156093. doi: 10.1016/j.phymed.2024.156093 39531934

[143] Chen LM Yang PP Al Haq AT Hwang PA Lai YC Weng YS . Oligo-fucoidan supplementation enhances the effect of olaparib on preventing metastasis and recurrence of triple-negative breast cancer in mice. J BioMed Sci. (2022) 29:70. doi: 10.1186/s12929-022-00855-6 36109724 PMC9479298

[144] Wang S Li J Xu S Wang N Pan B Yang B . Baohuoside I chemosensitises breast cancer to paclitaxel by suppressing extracellular vesicle/cxcl1 signal released from apoptotic cells. J Extracell Vesicles. (2024) 13:e12493. doi: 10.1002/jev2.12493 39051750 PMC11270583

[145] Meng Q He J Zhong L Zhao Y . Advances in the study of antitumour immunotherapy for newcastle disease virus. Int J Med Sci. (2021) 18:2294–302. doi: 10.7150/ijms.59185 33967605 PMC8100649

[146] Zhang WH Chen L Gao L Wu YM Jin ZC Chen JJ . Engineered macrophage membrane-mimicking nanodrugs activate cgas/sting pathway to reverse tumor immune suppression after incomplete radiofrequency ablation. J Nanobiotechnol. (2025) 24:55. doi: 10.1186/s12951-025-03889-8 41398588 PMC12821273

[147] de Carvalho TG Lara P Jorquera-Cordero C Aragão CFS de Santana Oliveira A Garcia VB . Inhibition of murine colorectal cancer metastasis by targeting m2-tam through stat3/nf-kb/akt signaling using macrophage 1-derived extracellular vesicles loaded with oxaliplatin, retinoic acid, and libidibia ferrea. Biomed Pharmacother = Biomed Pharmacotherapie. (2023) 168:115663. doi: 10.1016/j.biopha.2023.115663 37832408

[148] Mehta A Popplewell L Collins GP Smith SM Flinn IW Bartlett NL . Magrolimab plus rituximab in relapsed/refractory indolent non-hodgkin lymphoma: 3-year follow-up of a phase 1/2 trial. Blood Adv. (2024) 8:5855–63. doi: 10.1182/bloodadvances.2024013277 39213421 PMC11609520

[149] Jiménez I Carabia J Bobillo S Palacio C Abrisqueta P Pagès C . Repolarization of tumor infiltrating macrophages and increased survival in mouse primary cns lymphomas after xpo1 and btk inhibition. J Neuro-Oncol. (2020) 149:13–25. doi: 10.1007/s11060-020-03580-y 32691208 PMC7452938

[150] Zhang Z Wu Q Xie M Ye R Geng C Shi J . Layered double hydroxide-loaded si-NEAT1 regulates paclitaxel resistance and tumor-associated macrophage polarization in breast cancer by targeting miR-133b/PD-L1. Nan Fang Yi Ke Da Xue Xue Bao. (2025) 45:1718–31. doi: 10.12122/j.issn.1673-4254.2025.08.16 40916533 PMC12415571

[151] Xiang X Wang F Shen Y Dai S . Clever-1 blockade reprograms tams to overcome anti-pd-1 resistance in gastric cancer. J Immunother Cancer. (2025) 13:e013145. doi: 10.1136/jitc-2025-013145 41339106 PMC12682175

[152] Han R Guo H Shi J Zhao S Jia Y Liu X . Osimertinib in combination with anti-angiogenesis therapy presents a promising option for osimertinib-resistant non-small cell lung cancer. BMC Med. (2024) 22:174. doi: 10.1186/s12916-024-03389-w 38658988 PMC11040894

[153] Comunanza V Gigliotti C Lamba S Doronzo G Vallariello E Martin V . Dual vegfa/braf targeting boosts pd-1 blockade in melanoma through gm-csf-mediated infiltration of m1 macrophages. Mol Oncol. (2023) 17:1474–91. doi: 10.1002/1878-0261.13450 37183363 PMC10399721

[154] Tamura R Tanaka T Morimoto Y Kuranari Y Yamamoto Y Takei J . Alterations of the tumor microenvironment in glioblastoma following radiation and temozolomide with or without bevacizumab. Ann Trans Med. (2020) 8:297. doi: 10.21037/atm.2020.03.11 32355741 PMC7186631

[155] Wiegmans AP Ivanova E Naei VY Monkman J Fletcher J Mullally W . Poor patient outcome correlates with active engulfment of cytokeratin positive ctcs within cancer-associated monocyte population in lung cancer. Clin Exp Metastasis. (2024) 41:219–28. doi: 10.1007/s10585-024-10270-w 38416302 PMC11213738

[156] Pender A Titmuss E Pleasance ED Fan KY Pearson H Brown SD . Genome and transcriptome biomarkers of response to immune checkpoint inhibitors in advanced solid tumors. Clin Cancer Res. (2021) 27:202–12. doi: 10.1158/1078-0432.ccr-20-1163 33020056

[157] Li X Li Z Zhai J Zou B . A macrophage-derived 7-gene signature predicts prognosis and therapeutic response in hepatocellular carcinoma. IUBMB Life. (2025) 77:e70079. doi: 10.1002/iub.70079 41355397 PMC12683284

[158] Xue R Zhang Q Cao Q Kong R Xiang X Liu H . Liver tumour immune microenvironment subtypes and neutrophil heterogeneity. Nature. (2022) 612:141–7. doi: 10.1038/s41586-022-05400-x 36352227

[159] Chu X Tian Y Lv C . Decoding the spatiotemporal heterogeneity of tumor-associated macrophages. Mol Cancer. (2024) 23:150. doi: 10.1186/s12943-024-02064-1 39068459 PMC11282869

[160] Wang P Liu Y Wei L Wang J Wang J Du B . Development of a novel prognostic model for esophageal squamous cell carcinoma: insights into immune cell interactions and drug sensitivity. Cancer Invest. (2024) 42:243–59. doi: 10.1080/07357907.2024.2340576 38616306

[161] Lyu A Fan Z Clark M Lea A Luong D Setayesh A . Evolution of myeloid-mediated immunotherapy resistance in prostate cancer. Nature. (2025) 637:1207–17. doi: 10.1038/s41586-024-08290-3 39633050 PMC11779626

[162] Zhang C Li K Zhu H Cheng M Chen S Ling R . Itgb6 modulates resistance to anti-cd276 therapy in head and neck cancer by promoting pf4(+) macrophage infiltration. Nat Commun. (2024) 15:7077. doi: 10.1038/s41467-024-51096-0 39152118 PMC11329676

[163] Kamboj D Gupta P Basil MV Mohan A Guleria R Bhatnagar A . Improved mycobacterium tuberculosis clearance after the restoration of ifn-γ(+) tnf-α(+) cd4(+) t cells: impact of pd-1 inhibition in active tuberculosis patients. Eur J Immunol. (2020) 50:736–47. doi: 10.1002/eji.201948283 32113187

