# Supplementary material for: Molecular innate immune programs of tumor-associated macrophages in immune checkpoint blockade resistance: a staged framework from suppressive circuitry to translational bottlenecks
Source: Front Immunol. 2026 Aug 6;17:1881958. doi: 10.3389/fimmu.2026.1881958 (PMC13491056; doi:10.3389/fimmu.2026.1881958)
Supplement: Supplementary file 1 [file DataSheet1.docx]

Supplementary Material

# 1 Supplementary Data

Supplementary Material should be uploaded separately on submission. Please include any supplementary data, figures and/or tables.

Supplementary material is not typeset so please ensure that all information is clearly presented, the appropriate caption is included in the file and not in the manuscript, and that the style conforms to the rest of the article.

# 2 Supplementary Figures and Tables

For more information on Supplementary Material and for details on the different file types accepted, please see [here](https://www.frontiersin.org/guidelines/author-guidelines#supplementary-material).

## 2.1 Supplementary Figures

Supplementary Table S1. Representative TAM subpopulations and spatial programs associated with ICB resistance

| TAM subpopulation or spatial program | Tumor context | Main biological function | Relevance to ICB resistance | Ref. |
| --- | --- | --- | --- | --- |
| SPP1+ TAMs | Gastric cancer, TNBC, GBM, ccRCC | Promote stromal remodeling, immunosuppressive TME formation, and CD8+ T-cell dysfunction | Represent a recurrent suppressive TAM module associated with poor ICB response | [25–32] |
| C1q+ TAMs | Malignant pleural effusion and other suppressive TMEs | Support fatty acid metabolic reprogramming and PD-L1-associated immunosuppression | May define a metabolically suppressive TAM state relevant to checkpoint resistance | [33] |
| TREM2+ TAMs | Multiple solid tumors | Maintain immunosuppressive macrophage programs | TREM2 targeting enhances immunotherapy efficacy in experimental models | [34] |
| TREM-1+ TAMs | Hypoxic HCC niches | Promote Treg recruitment and local immune suppression | Contribute to anti-PD-L1 resistance in hypoxic tumor regions | [35] |
| TAM_C3 / Rps19–C5aR1-associated TAMs | PARP inhibitor-resistant breast cancer | Display anti-inflammatory and pro-tumoral gene programs | C5aR1 inhibition may selectively target therapy-resistant macrophage subsets | [36] |
| USP14+ TAMs | Anti-PD-1-resistant gastric cancer | Recruit MDSCs through the CXCL2 axis | Promote suppressive myeloid remodeling and anti-PD-1 resistance | [37] |
| CAF-associated TAM niches | Gastric cancer, TNBC, stromal-rich tumors | Integrate CAF-derived signals with suppressive macrophage programs | Support stromal–myeloid immune exclusion and resistance to ICB | [25,30,38] |
| Macrophage-coated or perivascular tumor niches | HCC, prostate cancer, vascular-rich tumors | Form physical or functional barriers to T-cell infiltration | Highlight the importance of spatial organization beyond TAM abundance alone | [39,40] |

Supplementary Table S2. Representative TAM education cues and suppressive resistance mechanisms

| Mechanistic category | Representative molecules or axes | Main effect on TAMs or immune cells | Resistance implication | Ref. |
| --- | --- | --- | --- | --- |
| Tumor-intrinsic TAM education | CREB3L1, PEAK1, LRP1, GPSM1 | Promote M2-like or suppressive macrophage polarization | Establish tumor-specific macrophage education programs that weaken ICB efficacy | [48,49,50,56] |
| Stromal-driven TAM education | LOXL2-high iCAFs, CAF-derived C3, SAA, TSG-6 | Drive suppressive TAM differentiation and stromal–myeloid coupling | Reinforce immune-excluding niches and reduce T-cell access | [5,25,51,90] |
| Cytokine-driven polarization | Tumor-derived IL-4, IL-4/IL-4R axis, IL-34 | Expand suppressive CD163+ or M2-like TAM states | Contribute to anti-PD-1 resistance and broader therapy resistance | [24,52,64–66] |
| Macrophage-intrinsic signaling | MALT1, FKBP51s, PERK+ macrophage programs | Maintain tolerant or suppressive macrophage states | Identify intracellular programs that may sustain TAM-mediated immune escape | [53,54,57] |
| Metabolic education | Lactate, acidosis/GPR65, methionine availability | Reprogram TAMs toward suppressive or M2-like states | Links metabolic stress to macrophage-mediated ICB resistance | [58–60] |
| Soluble suppressive circuits | Granulin, SPP1, CCL7, IL-33 | Exclude CD8+ T cells, impair T-cell function, or activate Tregs | Suggest that dominant soluble circuits should guide targeted combinations | [32,62,63,70] |
| Extracellular vesicle-mediated crosstalk | M2-EV miR-21-5p, tumor-derived exosomal PD-L1 | Promote T-cell exhaustion or systemic immunosuppression | Extend TAM-mediated resistance beyond local cytokine signaling | [68,69] |
| Checkpoint ligand expression | PD-L1, VISTA, Siglec-15, TIM-3-related programs, IDO-1 | Directly suppress T-cell activation or mark treatment failure | Requires evaluation of checkpoint ligand cellular source and spatial position | [71–84] |
| Metabolic suppression of effector immunity | Kynurenine–AHR, adenosine–A2A, UDP–P2Y6, arginine depletion | Impair T-cell fitness and reinforce suppressive macrophage states | Makes metabolism a structural driver of local immune failure | [85–89] |
| Impaired phagocytosis and antibody handling | CD47–SIRPα, CD24–Siglec-10/G, STC1, FcγR-mediated antibody stripping | Block macrophage-mediated clearance or reduce effective antibody engagement | Links innate immune evasion with reduced ICB activity | [91–96] |

Supplementary Table S3. Representative TAM-targeted therapeutic interventions

| Therapeutic category | Representative targets or approaches | Proposed mechanism | Translational consideration | Ref. |
| --- | --- | --- | --- | --- |
| TAM depletion or remodeling | CSF1/CSF1R inhibition | Reduces or remodels macrophage populations dependent on CSF1R signaling | May spare resistant TAM subsets and may also affect beneficial macrophage functions | [120,121] |
| Recruitment blockade | CCL2–CCR2, CCR2/5, CCR1-related axes | Limits monocyte or macrophage influx into tumors | Chemokine redundancy may limit monotherapy efficacy | [120,121,123] |
| PI3K/AKT/mTOR-related targeting | TYRO3, Akt, PI3Kγ, PI3Kγδ | Disrupts survival and metabolic signaling that stabilizes suppressive TAM states | Best applied in genotype- or therapy-defined settings | [61,124–126] |
| Tumor–TAM circuit disruption | CCL15–CCR1, CCL3–CCR1, circSDHAF2/ITGA5, GPSM1–MEIS3–CSF1 | Blocks reciprocal tumor–macrophage circuits that generate suppressive TAM states | Requires identification of the dominant tumor–myeloid circuit | [31,56,123,127] |
| Stress-response pathway targeting | GPR65, HPGDS, TREM-1 | Reverses acidosis-, prostaglandin-, or hypoxia-driven TAM suppression | May require combination with metabolic or stromal remodeling | [35,58,128] |
| Inflammatory signaling modulation | STAT3, NF-κB, PARP14, TNF/p65, p38 MAPK | Reprograms TAM inflammatory or suppressive signaling states | Context-dependent effects require functional monitoring | [129–134] |
| Innate immune activation | STING agonist delivery to FR-β+ TAMs | Induces IFNβ production and cytotoxic lymphocyte activation | Local delivery may reduce systemic inflammatory toxicity | [39] |
| Phagocytosis checkpoint targeting | CD47–SIRPα, CD24-related adaptive resistance | Restores macrophage-mediated tumor cell engulfment and antigen presentation | Toxicity and tumor-context specificity remain major barriers | [91–93] |
| Metabolic and epigenetic modulation | HDAC inhibition, 2-DG, APOC1 inhibition, methionine restriction, lncRNA modulation | Reprograms TAM metabolism or epigenetic state toward antitumor activity | Requires patient selection based on metabolism-dependent TAM resistance | [60,135–139] |
| Repurposed agents and delivery platforms | COX-2 inhibitors, honokiol, rimantadine, NDV, nanoparticles, engineered EVs, biomimetic nano decoys | Remodel macrophage polarization or deliver TAM-modulating agents locally | Should be presented as convergent TAM-reprogramming strategies rather than isolated drug lists | [83,121,140–147] |

Supplementary Table S4. Candidate clinical strategies and biomarkers for TAM-directed translation

| Strategy or biomarker | Main function | Clinical or translational relevance | Ref. |
| --- | --- | --- | --- |
| Anti-CCR2/5 therapy | Blocks monocyte recruitment into the TME | May reduce TAM replenishment, but efficacy can be limited by redundant chemokine pathways | [120,121] |
| CSF1/CSF1R inhibitors | Deplete or reprogram TAMs | Clinically attractive but constrained by TAM heterogeneity, compensatory pathways, and toxicity | [120,121] |
| Anti-CD47 therapy | Blocks the CD47–SIRPα “don’t eat me” axis | Promotes macrophage-mediated phagocytosis; clinical activity is most established in selected hematologic malignancies | [148] |
| Anti-CLEVER-1 therapy | Reprograms TAMs toward a pro-inflammatory phenotype | May enhance anti-PD-1 responsiveness by restoring macrophage antitumor function | [151] |
| Tumor mutation burden | Broad genomic biomarker for ICI response | Useful in selected settings but insufficient to capture TAM-specific resistance programs | [156] |
| Macrophage-related gene signatures | Seven-gene macrophage signature in HCC and other composite myeloid signatures | May stratify patients for ICB versus targeted therapy | [157] |
| Cancer-associated macrophage-like cells | Circulating phagocytic monocyte/macrophage-like cells | Provide a liquid-biopsy readout of myeloid activity and prognosis in NSCLC | [155] |
| Spatial profiling | Spatial transcriptomics, multiplex imaging, TAM–CAF–tumor neighborhood analysis | Identifies immune-excluding macrophage niches and supports spatially informed trial design | [25,38–40,159] |
